# Artificial Intelligence-Powered Materials Science

**DOI:** 10.1007/s40820-024-01634-8

**Published:** 2025-02-06

**Authors:** Xiaopeng Bai, Xingcai Zhang

**Affiliations:** 1 World Tea Organization, Cambridge, MA 02139 USA; 2 Department of Materials Science and Engineering, Stanford University, Stanford, CA 94305 USA; 3 Department of Mechanical Engineering, The University of Hong Kong, Hong Kong, 999077 People’s Republic of China; 4 Department of Physics, The Chinese University of Hong Kong, Shatin, Hong Kong, 999077 People’s Republic of China

**Keywords:** Artificial intelligence, Machine learning, Sustainable materials, Data-driven, Materials innovation

## Abstract

The advancement of materials has played a pivotal role in the advancement of human civilization, and the emergence of artificial intelligence (AI)-empowered materials science heralds a new era with substantial potential to tackle the escalating challenges related to energy, environment, and biomedical concerns in a sustainable manner. The exploration and development of sustainable materials are poised to assume a critical role in attaining technologically advanced solutions that are environmentally friendly, energy-efficient, and conducive to human well-being. This review provides a comprehensive overview of the current scholarly progress in artificial intelligence-powered materials science and its cutting-edge applications. We anticipate that AI technology will be extensively utilized in material research and development, thereby expediting the growth and implementation of novel materials. AI will serve as a catalyst for materials innovation, and in turn, advancements in materials innovation will further enhance the capabilities of AI and AI-powered materials science. Through the synergistic collaboration between AI and materials science, we stand to realize a future propelled by advanced AI-powered materials.

## Highlights

- A detailed exploration is provided of how artificial intelligence (AI) and machine learning techniques are applied across various aspects of materials science.
- Major challenges in AI-driven materials science are evaluated.
- Novel case studies are incorporated, demonstrating their impact on accelerating material development and discovery.

## Graphical abstract

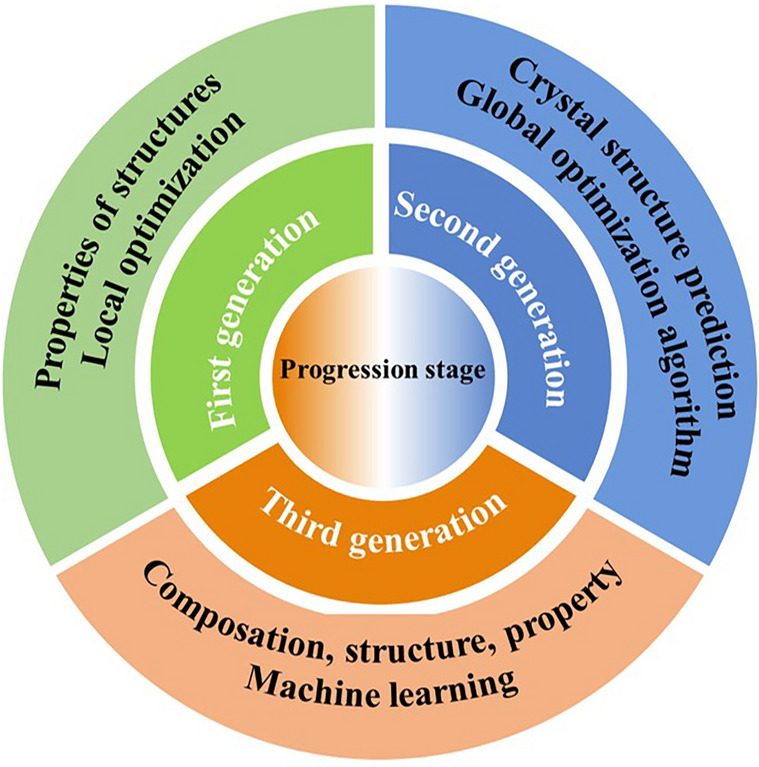

## Introduction

Material science has emerged as a pivotal nexus for the advancement and maturation of contemporary science and technology, assuming a foundational and pioneering role in their development. Each stride taken in material science theory exerts a catalytic influence on the innovation of materials technology and materials engineering. Noteworthy breakthroughs achieved in key material technologies have the potential to foster advancements across multiple scientific and technological domains. Furthermore, the advent of novel materials holds the prospect of instigating the inception of nascent industrial sectors.

The conventional model for material research and development primarily relies on scientific researchers who design experiments and continuously optimize experimental parameters in order to attain optimal materials. This process typically spans a duration of 10–20 years, requiring significant engineering efforts, extensive consumption of experimental materials, and substantial labor costs. These factors have posed substantial obstacles to meeting the demands for novel materials in twenty-first-century industrial development. However, with the advancements in information technology within the domain of material simulation, the trajectory of materials research and development has shifted from an experimental-driven paradigm to a computational-driven one [1]. Through the application of theoretical and computational simulations, promising candidate materials can be predicted, subsequently narrowing down the scope of experimental validation. This approach is currently extensively employed. Moreover, with the advent of AI, the present landscape of material research and development has progressively transitioned into a data-driven phase [2]. Drawing upon machine learning and data mining techniques, models are constructed based on substantial datasets to predict potential materials. This methodology is grounded in theoretical calculations, and the utilization of high-throughput computing systems enables the rapid acquisition of vast amounts of data. By leveraging artificial intelligence for screening and designing novel materials, the pace of material research and development is significantly enhanced, while costs are concurrently reduced.

## Artificial Intelligence and Machine Learning

### Artificial Intelligence

Artificial intelligence, commonly referred to as AI, encompasses a system that enables humans to emulate human cognition and behavior. AI entails the emulation of human intelligence in programmed machines, enabling them to replicate human-like thinking, actions, and task completion that were formerly exclusive to natural intelligence [1]. By harnessing data-driven AI technology [2], the capabilities of AI systems transcend those of natural or human intelligence in terms of speed, efficiency, and productivity. AI-based programs, such as Siri on Apple devices, possess the capacity to analyze and process data while emulating human cognitive abilities. To streamline the cycle of material research and development, artificial intelligence serves as a potent auxiliary tool that employs data sharing to predict and screen the physicochemical properties of advanced materials, thus expediting the synthesis and production of new materials. In essence, AI endeavors to imbue programs with enhanced human-like processing and task execution capabilities.

### Machine Learning

Machine learning, a subset of AI, utilizes data-driven techniques to solve specific tasks by learning from data and making predictions. It involves extracting knowledge and predictions from extensive datasets, eliminating the need for explicit programming. The core of machine learning lies in training machine learning models, which optimize their parameters by iteratively comparing them to actual values. This optimization process, which involves millions of iterations, aims to minimize the discrepancy between simulated and real-world scenarios. Machine learning algorithms play a significant role in assisting the design of novel materials as part of the broader field of AI. The workflow of machine learning in materials science primarily encompasses four steps: descriptor generation, model construction and verification, material prediction, and experimental validation. Descriptors capture specific material properties based on existing data, enabling the construction of nonlinear training models to predict the properties of new materials. This process circumvents the reliance on traditional physical knowledge. In comparison with conventional trial-and-error research methods, machine learning technology offers benefits such as low cost, high efficiency, shorter cycles, and scalability [3]. On the one hand, the field of materials science has generated vast amounts of data in the information age, establishing extensive databases. Given that machine learning's core statistical algorithms excel in processing and generalizing big data, it becomes possible to extract new insights from existing experimental data, explore intricate implicit relationships between various parameters, establish accurate prediction models, and leverage the full potential of experimental data. On the other hand, prevalent computational simulation methods used in computational materials science, such as first-principles calculations [4], molecular dynamics [5], and finite element simulation [6], often entail significant time and resource consumption while possessing inherent limitations. Material simulations aim to predict the properties of new materials based on existing data through mathematical modeling. Frequently, the relationships between input and output material properties exhibit complexity that traditional linear and nonlinear association methods struggle to handle. Machine learning, however, enables the identification of such relationships through modeling. Additionally, machine learning not only significantly reduces computing time but also expands the spatial and temporal scales of computational systems. For material design, the crucial step involves constructing an associative model that accurately captures the relationship between input material-specific features and properties of interest, based on a given dataset. Classical models heavily rely on physical perspectives and mechanisms, utilizing mathematical formulations to derive parameters, typically linear or slightly nonlinear, from existing reference data using conservation laws and thermodynamics. Machine learning takes a distinct approach, training models in flexible and often highly nonlinear forms based solely on available data, without dependence on physical principles or knowledge. In materials science, complex relationships frequently exist between a material's structure and the properties of interest, challenging traditional correlation methods. Consequently, machine learning methods have emerged as vital tools for predicting material properties, material screening, and optimal design [7–9]. Thus, data-driven approaches represent a crucial development direction for the future of materials science. This review aims to provide insights into the current progress and future prospects of AI-powered materials science. One area where AI has made significant contributions is in materials discovery and development. Traditionally, the process of discovering new materials involved a combination of empirical experimentation and theoretical calculations based on established physical principles. However, this approach is often time-consuming, expensive, and limited in its ability to explore the vast space of possible materials and their properties. AI-powered machine learning algorithms have revolutionized this process by enabling the rapid screening of materials and the prediction of their properties. The first step in applying machine learning to materials science is the generation of descriptors. Descriptors are numerical representations of specific material properties or features derived from existing data. These descriptors capture important characteristics of materials that can be used to train machine learning models. By utilizing descriptors, machine learning models can learn complex relationships between input features and material properties, allowing for accurate predictions of the properties of new materials. Once descriptors are generated, machine learning models are constructed and verified using training data. The models are trained by iteratively adjusting their parameters to minimize the error between predicted and actual values. This iterative optimization process, often involving millions of iterations, allows the models to learn from the data and improve their predictive performance. After the models are trained and verified, they can be used to predict the properties of new materials. This enables researchers to rapidly screen a large number of materials and identify those with desirable properties for specific applications. The predictions made by machine learning models can guide experimental efforts by suggesting promising materials that can be synthesized and tested in the laboratory. Experimental validation is a crucial step in the machine learning workflow. The predicted properties of materials need to be experimentally verified to ensure their accuracy and reliability. This iterative process of prediction and validation helps refine and improve the machine learning models, leading to more accurate predictions in subsequent iterations.

### Progression Stage of Materials Simulation

**Fig. 1:**
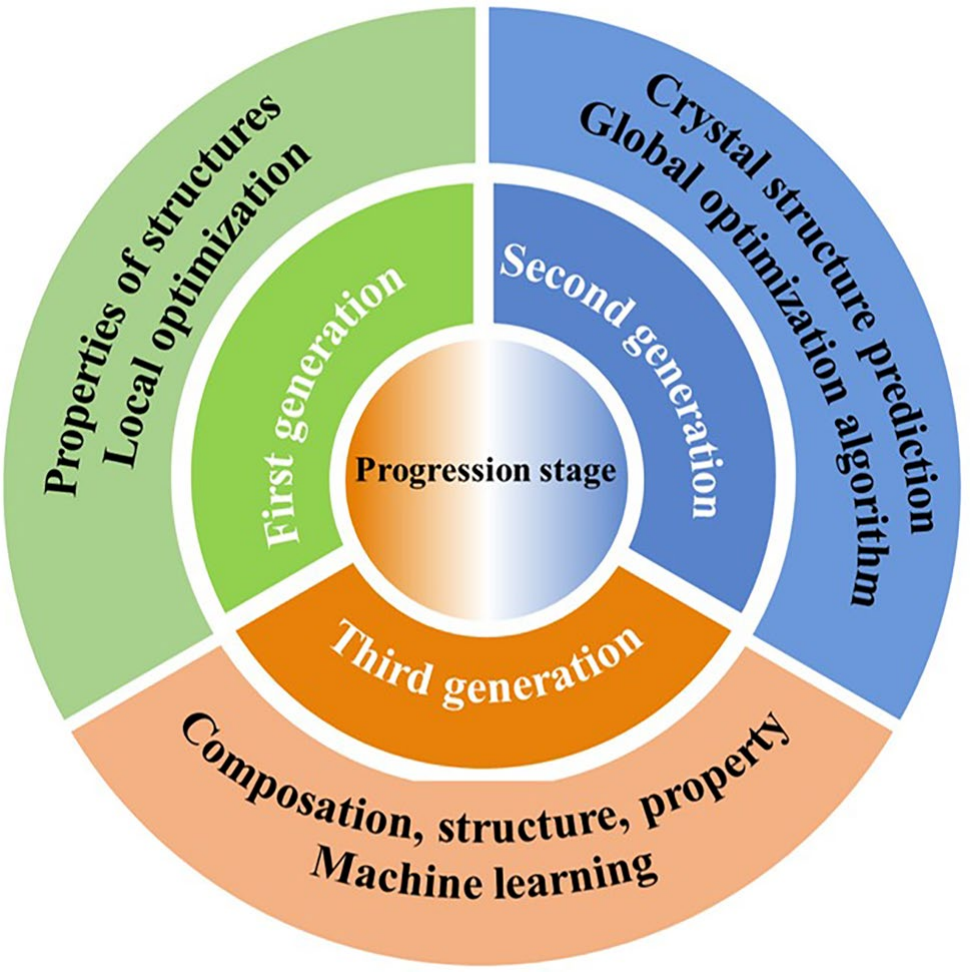
Evolutionary process of computational simulation for predicting material properties

The prediction of material properties through computational simulation has evolved across three generations (Fig. 1). The first generation involves calculating the physical properties of input structures, typically achieved by approximating the Schrödinger equation and employing local optimization techniques. The second generation focuses on predicting structures or combinations of structures based on the composition of input materials, utilizing global optimization algorithms. The third generation utilizes machine learning to predict compositions, structures, and properties of materials by leveraging experimental data (adequate data is essential for training suitable models) [10]. This data-driven computational approach necessitates the establishment of a novel infrastructure. It becomes imperative to develop a comprehensive innovation platform with data at its core, integrating "high-throughput experiment," "high-throughput calculation," and "material-data platform."

### Development Process of New AI Materials

The development process of novel AI materials encompasses three distinct stages: data acquisition through the characterization and computational platform of existing materials, data analysis utilizing AI data models, and the generation of new materials based on the identified data characteristics.

### Machine Learning Databases

As widely acknowledged, data collection is typically achieved through experimental means. However, the substantial volume of experimental data resources necessitated often presents a bottleneck for the development of machine learning. Currently, existing databases are enumerated in Table 1.

**Table 1:** Databases commonly used in the material field

Databases	Material category	Material properties
ZINC [11], ChEMBL [12], GDB-13 [13], GDB-17 [14]	Molecules	Low melting and boiling points
International Crystal Structure Database (ICSD) [15], Open Quantum Materials Database (OQMD) [16]	Inorganic crystals	High thermal stability, high modulus
Harvard Clean Energy Project [17]	Organic solar-absorber materials	Made up of polymers or π-bonded molecules
CoRE MOF [18]	Over 4000 metal–organic framework materials	Porous structures
Cambridge Structure Database [19]	Organic and metal–organic crystal structures	3D structures contain a wide range of organic, metal–organic, organometallic molecules
Computational Materials Repository [20]	Electronic-structure codes	The state of motion of electrons in an electrostatic field
PubChem [21]	Biological activities of small molecules	Small molecules with less than 100 atoms and 1,000 bonds
Computational 2D Materials [22]	2D Materials	Crystalline solids consisting of a single layer of atoms

The characterization and application scopes of these machine learning databases are shown in below.

#### ZINC8

ZINC8 is a machine learning database specifically designed to furnish chemical compound information. It encompasses an extensive assortment of virtual compounds, thereby affording researchers the opportunity to explore a wide range of chemical space and gain access to potential drug candidates. By integrating diverse sources of chemical and biological data, this database facilitates virtual screening and leads optimization processes pertinent to drug discovery projects. The expansive library of compounds offered by ZINC8 proves invaluable to researchers by furnishing them with invaluable molecular insights, thereby aiding the development of novel therapeutic interventions.

#### ChEMBL

ChEMBL represents a comprehensive machine learning database that concentrates on the storage and retrieval of bioactivity data for small molecule drug-like compounds. This database delivers meticulous information regarding the interaction between these compounds and their corresponding targets, such as proteins and enzymes. By enabling scientists to investigate structure–activity relationships (SAR) and undertake target identification and validation endeavors, ChEMBL emerges as an indispensable resource in the realm of drug discovery and development. Moreover, owing to its access to large-scale bioactivity data, this database facilitates the exploration of chemical space and expedites the design of novel pharmaceutical agents.

#### GDB-13 and GDB-17

GDB-13 and GDB-17 are machine learning databases that focus on the enumeration and exploration of small organic molecules. These databases provide exhaustive and methodical collections of chemically feasible compounds based on predefined structural rules and constraints. While GDB-13 encompasses around 977 million unique molecules, GDB-17 further expands this collection to approximately 166 billion molecules. These databases prove instrumental in virtual screening endeavors, de novo drug design processes, and the comprehensive investigation of chemical space in drug discovery and materials science.

#### International Crystal Structure Database

The International Crystal Structure Database (ICSD) serves as a machine learning database housing an extensive compilation of experimentally determined crystal structures. Researchers can gain access to precise and dependable crystallographic data, including atomic positions, unit cell parameters, and other essential structural details. Given its comprehensive nature, the ICSD emerges as an invaluable resource for crystallographers, material scientists, and chemists engaged in structure determination, crystallographic analysis, and the study of solid-state properties of materials.

#### Open Quantum Materials Database

The Open Quantum Materials Database (OQMD) is a machine learning database specializing in materials informatics and quantum mechanical calculations. Researchers are granted access to an extensive compendium of calculated properties pertaining to a broad range of inorganic compounds, including crystal structures, formation energies, electronic band structures, and thermodynamic properties. The OQMD facilitates high-throughput materials screening, prediction of novel materials, and the exploration of structure–property relationships employing quantum mechanical methods.

#### Harvard Clean Energy Project

The Harvard Clean Energy Project (CEP) functions as a machine learning database concentrating on the discovery and design of organic materials tailored for solar energy applications. By integrating computational methods, machine learning algorithms, and high-throughput screening techniques, the CEP aims to identify promising materials exhibiting desirable electronic and photovoltaic properties. Consequently, the CEP database expedites the exploration of organic materials suitable for photovoltaic purposes, thereby enabling researchers to hasten the development of efficient and cost-effective solar energy technologies.

#### CoRE MOF

CoRE MOF is a machine learning database that specializes in metal–organic frameworks (MOFs). MOFs are porous materials comprising metal ions or clusters coordinated with organic ligands, and they find diverse applications in gas storage, separation, and catalysis. The CoRE MOF database offers researchers access to a comprehensive compilation of experimentally characterized MOFs, encompassing their structures, porosity data, and adsorption properties. This database facilitates the discovery and design of new MOFs for various applications by enabling in-depth analysis of structure–property relationships.

#### Cambridge Structure Database

The Cambridge Structure Database (CSD) functions as a machine learning database that focuses on small organic and metal–organic crystal structures. It encompasses an extensive collection of experimentally determined crystal structures, providing atomic coordinates and crystallographic data for analysis. The CSD proves to be an invaluable resource for chemists and crystallographers, offering a wealth of structural information that can be utilized to study molecular conformations, intermolecular interactions, and crystal packing arrangements. Researchers can delve into the CSD to gain insights into chemical bonding, supramolecular assemblies, and the intricate relationship between structure and properties across a wide range of organic and metal–organic compounds.

#### Computational Materials Repository

The Computational Materials Repository (CMR) serves as a machine learning database that focuses on storing and sharing data related to computational materials science. It encompasses a wide range of material properties, including crystal structures, electronic band structures, thermodynamic properties, and mechanical properties. The CMR operates as a collaborative platform where researchers can share their simulation data, validate computational models, and compare results across different materials systems. This database fosters the development of new materials models, facilitates benchmarking of computational methods, and advances materials discovery and design through data-driven approaches.

#### PubChem

PubChem is a comprehensive machine learning database maintained by the National Center for Biotechnology Information (NCBI). It provides extensive information on the biological activities, chemical structures, and properties of small molecules. PubChem integrates data from various sources, including chemical literature, high-throughput screening experiments, and computational predictions. This database serves as a valuable resource for researchers engaged in drug discovery, chemical biology, and toxicology, allowing them to explore chemical space, identify potential drug targets, and analyze the biological effects of small molecules.

#### Computational 2D Materials

The Computational 2D Materials database focuses specifically on 2D materials, which are ultrathin layers of materials with distinctive electronic, optical, and mechanical properties. This database offers researchers access to computational models and simulations for various 2D materials, including graphene, transition metal dichalcogenides (TMDs), and other layered materials. It enables the exploration and prediction of 2D material properties such as electronic band structures, phonon dispersions, and optical response, thereby aiding in the design and optimization of novel 2D materials for applications in electronics, optoelectronics, and energy storage.

In conclusion, these machine learning databases play indispensable roles in advancing various academic fields, including drug discovery, crystallography, and computational modeling. They provide researchers with access to diverse chemical and materials data, thereby enabling them to explore chemical space, analyze structure–activity relationships, design new materials, and accelerate the discovery and development of innovative solutions in their respective domains.

### Machine Learning Algorithm Models

Upon gathering the requisite data, researchers must undertake the process of converting the data into a digital format comprehensible to machines. Open-source AI frameworks like TensorFlow, Keras, and Scikit-Learn are essential tools for developing machine learning models (Table 2). They offer flexibility and customization, making it possible to implement complex algorithms and cater to specific requirements. These frameworks benefit from large developer communities, encouraging collaboration, knowledge sharing, and the development of pre-built models and extensions. They are widely used in research, education, and practical applications, and they enable the deployment of models across various platforms. Consequently, numerous machine learning tools have been devised [22], as illustrated in Table 3.

**Table 2:** Open-source AI frameworks used in material science

Open-source AI frameworks	Characterization
TensorFlow	Building and training various machine learning and deep learning models
Keras	Open-source high-level neural networks API
Scikit-Learn	Wide range of tools for data preprocessing, classification, regression, clustering, dimensionality reduction
PyTorch	Dynamic computation graph
Caffe	Speed and efficiency in training deep neural networks

**Table 3:** Machine learning algorithm models used in material science

Algorithm model	Examples in materials science	Learning mode
C4.5 [23]	Analysis of the causes of coffee defects by decision tree [24]	Supervised learning
Naive Bayes [25]	Classification of metal binders [26]	
SVM [27]	Material monitoring and defect diagnosis [28] Prediction of rock brittleness [29]	
KNN [30]	Prediction of process parameters of reinforced metal casting [31] Analysis of welding modeling of different materials [32]	
Adaboost [33]	Temperature compensation of silicon piezoresistive pressure sensor [34]	
Cart [35]	Differential diagnosis of mucosanase [36]	
EM [37]	Estimation of dose distribution from positron [38] emitter distribution combined with filtering	Unsupervised learning
K-Means [39]	Structural texture similarity recognition of materials Establishment of parametric homogenized crystal plasticity model of single crystal Ni-base superalloy [40]	
COMBO [41]	Determining the atomic structure of crystal interfaces	
AFLOW-ML [42]	Retrieve predictions of electrical, thermal and mechanical properties	

Machine learning relies on various algorithms to tackle data problems, as there is no universally applicable algorithm that can address all types of problems. The choice of algorithm depends on the specific problem at hand. Machine learning algorithms can be categorized into supervised learning, unsupervised learning, and semi-supervised learning. To address the need for a more structured breakdown of AI techniques and their relevance to materials science, we have included a detailed comparative analysis of ML algorithms and deep learning (DL) models. ML algorithms such as support vector machines (SVMs) and decision trees excel in structured datasets with lower computational demands, making them suitable for tasks like defect detection and material classification. In contrast, DL models, including artificial neural networks (ANNs), are more effective for complex, high-dimensional data, such as predicting structure–property relationships or optimizing nanoporous materials. However, DL models require significantly more data and computational resources, making their deployment in resource-limited settings more challenging. Furthermore, critical challenges associated with AI integration, such as data standardization, model interpretability, and computational efficiency, are evaluated. For instance, ensuring standardized, high-quality datasets is vital for reproducibility, while explainable AI methods are crucial for aligning predictions with physical principles. Computational scalability, particularly for DL models, requires advances in high-performance computing and algorithm optimization to address the growing demands of training complex models.

Within supervised learning, examples include logistic regression and feedforward neural networks. Several commonly used learning algorithm models are described below. Decision tree (DT) is a versatile machine learning algorithm that is particularly well-suited for handling datasets with missing attribute values. By employing a hierarchical structure of nodes and branches, DT can efficiently process large-scale datasets within a relatively short time frame. However, one notable limitation of DT is its susceptibility to overfitting, which occurs when the model excessively captures noise or irrelevant patterns in the training data, leading to poor generalization on unseen data. Additionally, DT assumes that attributes are independent of each other, thereby neglecting potential correlations that exist among the data. This assumption can limit its ability to accurately capture complex relationships and interactions between variables. Despite these limitations, DT remains a valuable tool in machine learning, and various techniques, such as pruning and ensemble methods, have been developed to mitigate overfitting and enhance its performance in real-world applications. C4.5 is a well-known DT algorithm, with the "C" denoting its implementation in the C programming language and "4.5" indicating the specific version. It offers the advantage of generalizing relatively quickly and often achieving high precision. It is suitable for handling samples with missing attributes and can produce efficient results for large-scale data sources within a short timeframe. However, it is prone to overfitting and disregards the interrelation between data. Naive Bayesian: Bayesian classification is a general term encompassing a class of classification algorithms that leverage Bayesian theorem. Naive Bayes is the simplest classification method within this category, commonly employed for classification tasks involving multiple attributes. Support vector machine (SVM): SVM can handle the interaction of nonlinear features without relying on the entire dataset, enhancing its generalization ability and enabling the solution of high-dimensional problems. However, its efficiency decreases with an excessive number of samples due to reduced sensitivity to data. Linear regression (LR): LR is suitable for simple regression problems. It employs the least squares method to optimize the error function in the form of gradient descent. LR offers simplicity and fast training speed but cannot effectively fit nonlinear data. LR is well-suited for classification problems, providing fast calculation speed. It can be combined with regularization models to tackle specific challenges. K-Nearest neighbor (KNN) is a widely used machine learning algorithm that can be applied to various problem domains, encompassing both regression and classification tasks. In regression, KNN predicts the continuous values of a target variable by considering the average or weighted average of the values of its k-nearest neighbors. This allows KNN to capture nonlinear relationships between input features and the target variable. For classification tasks, KNN assigns a class label to a data point based on the majority class labels of its k-nearest neighbors. This flexible nature of KNN makes it suitable for addressing nonlinear classification problems, where decision boundaries are not linearly separable. By leveraging the distances and similarities between instances, KNN provides an effective and adaptable approach for analyzing complex datasets and making predictions in both regression and classification scenarios. Kernel ridge regression (KRR) is a powerful machine learning technique commonly applied in regression analysis and the prediction of material properties. It has gained popularity in the field of materials science, particularly for tasks such as predicting band gap values and synthesis enthalpy (energy) of materials. KRR combines the concepts of kernel methods and ridge regression to capture complex nonlinear relationships between input features and target variables. By utilizing a kernel function, KRR can implicitly map the input data into a higher-dimensional feature space, allowing for more flexible and accurate modeling. Moreover, KRR incorporates a regularization term, known as the ridge penalty, which helps prevent overfitting and improves generalization performance. The versatility and effectiveness of KRR make it a valuable tool for materials researchers in predicting and understanding various material properties, aiding in the design and development of new materials with desired characteristics. Artificial neural network (ANN) is a computational model inspired by the structure and functioning of biological neural networks. It has been widely utilized in materials science research to investigate and analyze an extensive range of crystal structures. By leveraging ANN, researchers can efficiently explore the relationship between the structure and properties of materials, particularly in the context of predicting electronic properties using small molecule force fields. ANN's ability to learn complex patterns and relationships from input data enables it to uncover valuable insights and visualize the structure–property relationship. Through training on a diverse dataset of crystal structures and their corresponding electronic properties, ANN can capture the underlying patterns and generalize this knowledge to make predictions for new materials [43]. This approach offers a powerful means of accelerating the discovery and design of materials with specific electronic properties, contributing to advancements in fields such as electronic devices, energy storage, and catalysis.

Unsupervised learning differs from supervised learning as it lacks correct answers or a teacher. Algorithms in unsupervised learning are designed to autonomously discover and reveal interesting structures within data. Unsupervised learning algorithms extract fewer features from the data, and when new data is introduced, these algorithms employ previously learned features to identify data categories. Clustering and feature reduction are the main applications of unsupervised learning. The k-means algorithm derives its name from its objective of creating k distinct and non-overlapping subgroups. Its goal is to maximize the similarity of data points within clusters while ensuring differentiation between clusters. The center of a cluster is determined by the average values of its data points. The algorithm seeks to minimize the squared distance between a data point and the centroid of its assigned cluster. Since there is no ground truth to evaluate the clustering algorithm's output against true labels, the primary aim is to explore the underlying data structure by partitioning it into distinct subgroups. Auto-encoder (AE) is a neural network architecture that leverages layer-by-layer unsupervised learning to compress input data. The AE consists of an encoder network that maps the input data to a lower-dimensional representation, and a decoder network that reconstructs the original data from the compressed representation. Through this compression and reconstruction process, the AE learns to capture the salient features and patterns present in the input data. After the unsupervised pre-training phase, the AE can further fine-tune its parameters through supervised learning, where the network is trained using labeled data to perform specific tasks such as classification or regression. This two-step learning approach of AE, combining unsupervised pre-training and supervised fine-tuning, enables the network to effectively learn meaningful representations from the data and enhance its predictive capabilities. AE has found numerous applications in various domains, including image recognition, anomaly detection, and data compression.

Semi-supervised learning is a machine learning algorithm utilized for datasets that comprise a mixture of labeled and unlabeled data. Unlabeled data typically correspond to a specific category within the labeled data and do not belong to multiple categories. The labels associated with the labeled data are assumed to be correct. Semi-supervised learning often leverages a small amount of labeled data alongside a larger volume of unlabeled data to address the challenge of acquiring a substantial labeled dataset. Some generative models, such as generative adversarial networks (GANs) and variational autoencoders (VAEs), can be used for semi-supervised learning. These models learn a latent representation of the data and can leverage both labeled and unlabeled instances to improve the quality of the learned representation and subsequent classification. Transductive support vector machine (TSVM) is an extension of traditional support vector machines (SVM) that incorporates unlabeled data into the decision boundary estimation process. It aims to find a decision boundary that separates labeled instances and unlabeled instances while maximizing the margin.

These classical algorithms hold significant potential for application in material information mining. However, further development of open-source algorithms is required to facilitate the wider adoption of machine learning-driven materials. The success and adoption of machine learning solutions and applications primarily depend on the effectiveness of both data and algorithms. If data suffers from poor representation, low correlation, or insufficient volume, the results obtained from machine learning models based on such data may become inaccurate. Thus, the validity of the data is crucial, in addition to selecting the appropriate machine learning model.

To ensure the validity and reliability of machine learning-driven materials, it is essential to address the shortcomings of data representation, correlation, and volume. Improving data quality and addressing these limitations will enhance the effectiveness of machine learning models. Furthermore, the development of open-source algorithms should be prioritized to facilitate broader accessibility and utilization of machine learning approaches in materials science. The widespread adoption of machine learning solutions and applications relies on the synergy between high-quality data and advanced algorithms. As researchers continue to refine and optimize these aspects, the potential for discovering valuable insights and accelerating materials research through machine learning will be further realized.

## Artificial Intelligence-Powered Materials

**Fig. 2:**
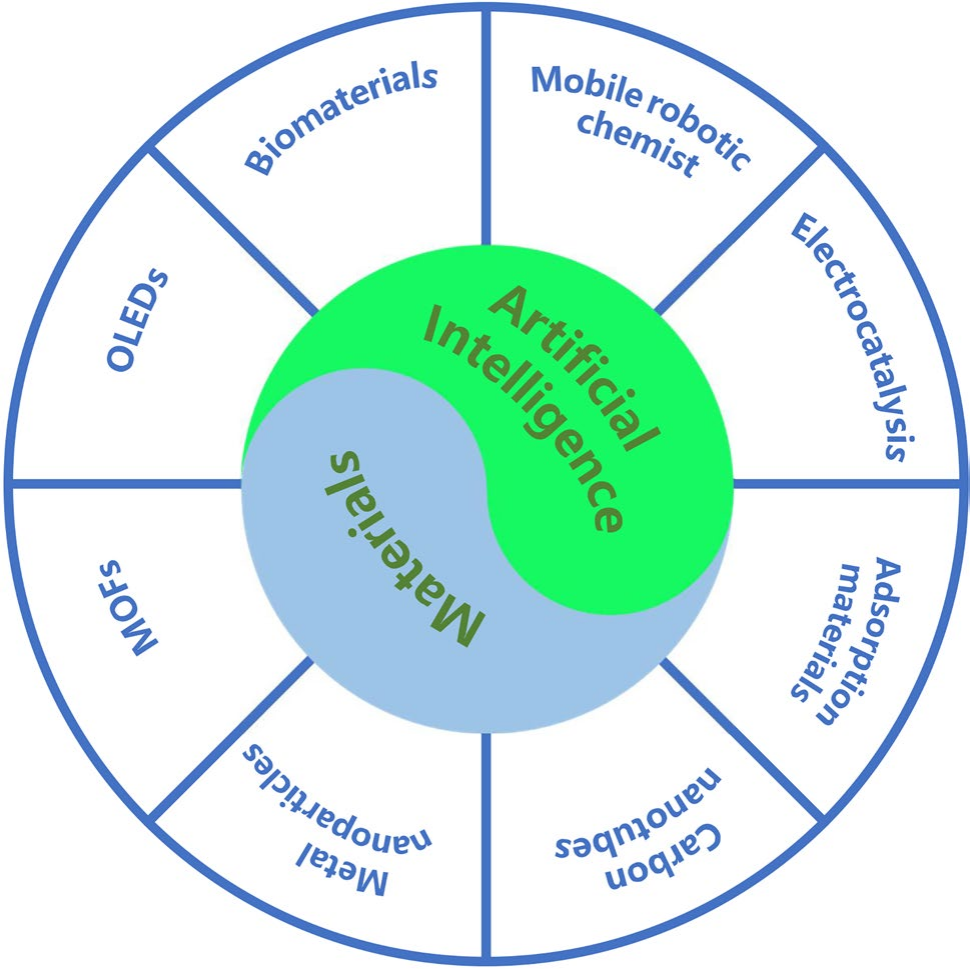
Artificial intelligence-powered materials

In recent years, the field of artificial intelligence has experienced a surge in interest pertaining to the innovation and investigation of novel materials, giving rise to extensive research endeavors and comprehensive documentation concerning various materials that have been engineered with the aid of artificial intelligence, as depicted in Fig. 2. These materials can be broadly categorized into three major classes, namely carbon-based functional materials, inorganic materials, and hybrid materials. Organic materials encompass a variety of substances, such as carbon nanotubes [44] and organic light-emitting diodes [45]. On the other hand, inorganic materials constitute another class, which includes materials like noble metal nanoparticles [46, 47] and two-dimensional materials [48, 49]. A noteworthy example of hybrid materials is MOFs [50, 51]. These materials exhibit versatile applicability across a wide spectrum of domains, encompassing organic field-effect transistors [52], micropattern manufacturing [53, 54], medical diagnosis [55], image processing [56], biomedicine [57, 58], the field of intelligent robotics [59], electrocatalysis [60], etc. In the following sections, we provide detailed insights into selected examples from this diverse range of materials. Upon analyzing the material's applications using AI, we categorize them into the following groups: materials discovery, property prediction, optimization and design, and process simulation and manufacturing.

### Materials Discovery

#### Carbon Nanotube

Carbon nanotubes possess high strength, stiffness, excellent electrical and thermal conductivity, and have found extensive applications in nanoelectronics, conductive cables, and biological and chemical sensors [61]. However, the synthesis of carbon nanotubes encounters challenges such as defects and low purity, primarily due to their sensitivity to experimental conditions such as temperature and pressure. Traditional methods of synthesis rely heavily on trial-and-error experimentation, which is time-consuming and inefficient. Here, AI plays a transformative role by drastically improving the synthesis process. In the study by Nikolaev et al., AI was used to design an innovative system called the artificial intelligence-based autonomous research system (ARES) [44]. ARES employs a machine learning platform to autonomously conduct experiments and optimize the synthesis of single-walled carbon nanotubes. The key advantage of this approach lies in its iterative closed-loop system, where the AI learns from each experiment, updates the database, and adjusts the experimental conditions for the next round of testing. This self-adjusting process allows the system to optimize CNT synthesis far more quickly than traditional manual methods.

##### How AI Accelerates the Discovery Process?

*Automated Experimental Design*: Once the initial database is created, ARES conducts experiments autonomously, generating new experimental parameters and adjusting conditions based on previous results. It performs over 600 experiments in an automated and iterative manner, speeding up the process compared to human researchers.

*Continuous Learning*: ARES uses a growing database of experimental results to refine its predictions. As more experiments are conducted, the discrepancy between the predicted and actual growth rates of the CNTs becomes smaller, indicating that the system is improving its understanding and predictions over time.

*Exploring Complex Parameter Spaces*: AI allows for the exploration of a wide range of experimental conditions that might be too complex or impractical for manual experimentation. With machine learning, ARES can navigate multidimensional parameter spaces, identifying optimal conditions for CNT synthesis much faster than traditional methods.

*In Situ* *Detection and Characterization*: ARES incorporates in situ detection and characterization to monitor CNT growth in real time. This real-time feedback loop allows for immediate adjustments, ensuring better synthesis outcomes.

**Fig. 3:**
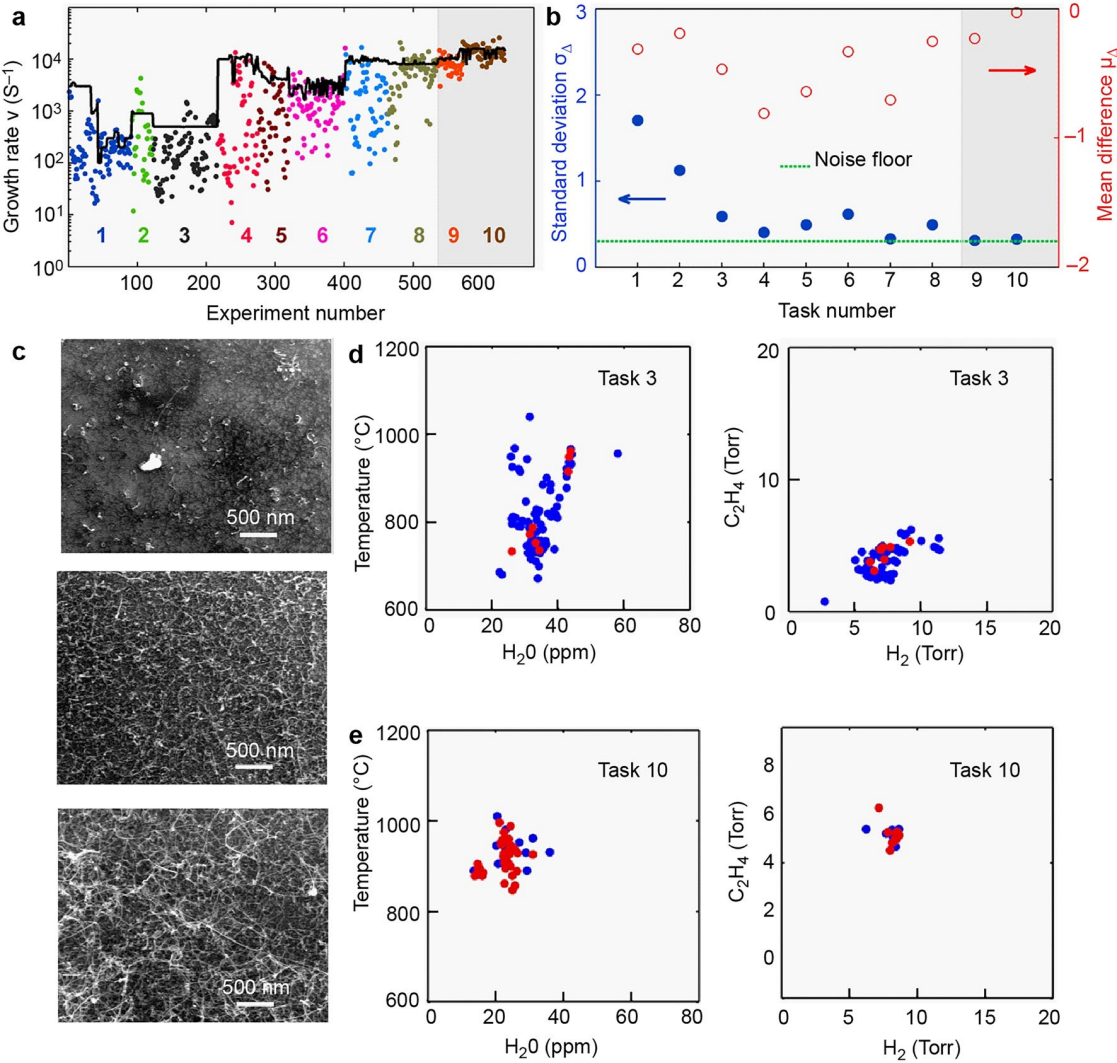
ARES for research on the synthesis of single-walled carbon nanotubes. **a** Comparison of the experimental growth rate and the predicted growth rate of ARES. **b** Convergence is assessed by quantifying the disparity between the experimentally observed growth rate and the predicted growth rate. **c** Carbon nanotubes synthesized based on simulated experimental parameters. **d** Model was trained using a limited and sparse dataset, resulting in a broad spectrum of parameter selections. **e** The model's dataset is three times larger than that depicted in Fig. 3d, leading to a narrower discrepancy between predictions and experimental outcomes. Reproduced with permission from Ref. [44]. Copyright 2016, The Author(s)

*Increased Speed and Efficiency*: As ARES grows its database and conducts more experiments, the system becomes increasingly accurate, reducing the number of trials needed to achieve desired results. In the study, it was shown that after a large dataset of experiments, ARES could predict and simulate conditions that resulted in the successful synthesis of CNTs in up to 68% of cases, a significant improvement over a sparse dataset where success was only achieved in 8% of experiments.This approach is orders of magnitude faster than traditional manual experiments and presents novel opportunities for synthesizing other materials using sophisticated techniques. Figure 3a depicts a comparison between the experimental growth rate and the predicted growth rate of ARES. As the number of experiments conducted by ARES increases from 0 to 600, the discrepancy between the experimental and predicted growth rates diminishes. The convergence is quantified by the difference between the experimental and predicted growth rates (Fig. 3b). It is evident that as the number of experiments increases, the difference gradually approaches zero. The carbon nanotubes grown according to the simulated experimental parameters are illustrated in Fig. 3c, exhibiting growth rates proportional to those observed in the simulated experiments (500, 3000, and 16,000 s^−1^). Importantly, when the model was trained using a limited and sparse dataset, a wide range of parameter choices resulted (Fig. 3d), and only 8% of experiments achieved the desired outcome. In Fig. 3e, with a dataset three times larger than that in Fig. 3d, the gap between predictions and experiments narrows (68% of experiments achieve the goal). This outcome demonstrates ARES's ability to simulate and explore complex multidimensional parameter spaces. The work also underscores the challenges and limitations of employing machine learning in materials research, such as the requirement for high-quality data and the potential for model overfitting. The authors emphasize the importance of combining domain knowledge and physical intuition with machine learning to ensure interpretable and reliable results. Overall, the paper provides a compelling demonstration of machine learning's potential to revolutionize materials research and facilitate new discoveries across various fields. As machine learning algorithms continue to advance and new data sources become available, autonomous materials research is expected to become increasingly prevalent and impactful in the future.

#### Organic Light-Emitting Diodes

The light-emitting layers in light-emitting diode (LED) devices are typically composed of electroluminescent molecules, and organic LEDs (OLEDs) are extensively employed in small displays due to their high efficiency and color rendering capabilities. However, OLEDs currently face challenges such as expensive material costs, low efficiency, and poor stability. To address these issues and eliminate the need for heavy atoms like iridium, a promising strategy is thermal activation delayed fluorescence (TADF) [62], which offers an efficient and cost-effective approach for OLED technology. By combining computational quantum chemistry, machine learning, organic synthesis, device fabrication, testing, and collaboration with industrial partners, it is possible to screen potential novel TADF materials. Through the exploration of 1.6 million molecules and the screening of over 400,000 molecules using transient density functional theory, machine learning techniques have facilitated the discovery of thousands of promising new organic light-emitting diode molecules in the visible spectrum [45].

### Property Prediction

#### Multimetallic Materials

**Fig. 4:**
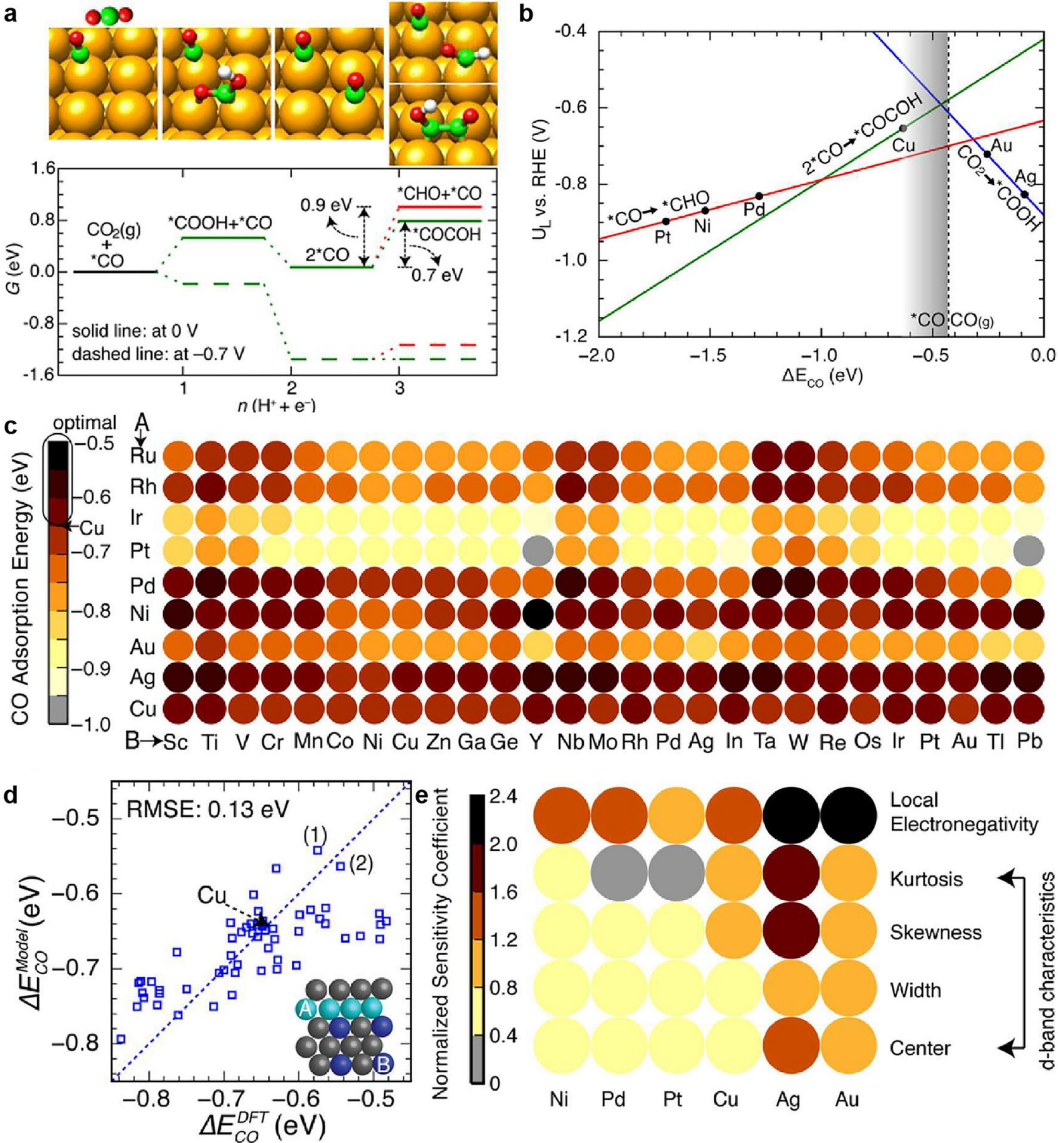
Machine learning-enhanced chemisorption model for CO_2_ electroreduction catalyst screening. **a** Most favorable free energy pathways for C_1_ and C_2_ species for CO_2_ electroreduction on Cu(100) at 0 and − 0.7 V with RHE. **b** Theoretical limiting potentials of C_1_ and C_2_ pathways for the electroreduction of CO_2_ as a function of the reactivity descriptor, the adsorption energy of CO. **c** Rational screening of CO adsorption energies on second-generation core–shell alloy surfaces (Cu_3_B-A@Cu ML) using the developed neural network model. **d** Parity plot illustrating the comparison of CO adsorption energies on selected Cu monolayer alloys calculated by the neural network model and DFT. **e** Normalized sensitivity coefficients obtained by analyzing the response of the network to perturbations of the input features. Reproduced with permission from Ref. [60]. Copyright 2015 Royal Society of Chemistry

Bimetallic or multimetallic materials have been extensively explored for catalyzing chemical and electrochemical reactions, as they can harness the synergistic effects of alloyed metal species to achieve new physicochemical properties on the surface [63–67]. However, the discovery of suitable alloy materials through high-throughput experiments and quantum chemical calculations is a time-consuming and expensive process. To address this challenge, AI-driven models have been increasingly utilized for property prediction to optimize material discovery and performance. For example, predicting physical, chemical, and mechanical properties such as adsorption energy, catalytic activity, and selectivity has proven crucial for identifying effective CO_2_ electroreduction catalysts. Currently, machine learning techniques, particularly ANNs, have shown significant promise in predicting these properties quickly and accurately, thereby facilitating the screening of alloy candidates. Among the transition metals, copper (Cu) exhibits remarkable electroreduction activity toward CO_2_, although the products of its reduction depend on the geometric arrangement of surface metal atoms. Cu(100), for example, shows high selectivity toward C_2_ species such as ethylene and ethanol, with low overpotentials (~ 0.8 V). Therefore, the design of 100-capped bimetallic or multimetallic materials is of great significance for enhancing the efficiency of CO_2_ reduction to C_2_ species. Ma et al. proposed a machine-learning-enhanced chemisorption model for screening CO_2_ electroreduction catalysts, enabling fast and accurate prediction of the surface reactivity of metal alloys across a wide chemical space [60]. Figure 4a illustrates the most favorable free energy pathways for C_1_ and C_2_ species during CO_2_ electroreduction on Cu(100) at 0 and − 0.7 V (vs. RHE), with snapshots of intermediate geometries depicted at the top. The authors calculated the theoretical limiting potentials of the C_1_ and C_2_ pathways for CO_2_ electroreduction as a function of the CO adsorption energy, a reactivity descriptor, as shown in Fig. 4b. The calculated CO adsorption energies (− 0.63 eV) on 1/8 monolayer (ML) of Cu(100) are in good agreement with the deduced measurements (− 0.66 eV) obtained from 0 to 1/8 ML *CO chemisorption differential heat measurements using the Perdew–Burke–Ernzerhof (PBE) exchange correlation function prediction. These findings confirm the excellent CO_2_ reducing activity of Cu among the transition metals. The construction of predictive models that relate the surface reactivity of metal sites to their electronic properties is a challenging task. However, with the availability of increasingly large materials databases, machine learning methods have emerged as a powerful solution, allowing complex physical interactions to be mapped onto statistical models. To achieve this, various input characteristics of the alloy surface and the corresponding CO adsorption energy data must be obtained in advance. These characteristics include the spatial extent of the metal *d* orbitals [68], the square of the adsorbate–metal interatomic *d* coupling matrix element, work function, atomic radius, ionization potential, electron affinity, and Pauling electronegativity. A nonlinear mapping between material characteristics and CO adsorption energy is established using an artificial neural network model. It is anticipated that numerous alloys will exhibit the desired CO binding energy, which is 0–0.2 eV weaker than CO adsorption on Cu(100). Employing a neural network model requires negligible CPU time, whereas performing standard density functional theory (DFT) calculations on hundreds of computers would take weeks or longer. Figure 4c illustrates the rational screening of CO adsorption energies on Cu3B-A@Cu monolayer surfaces using the neural network model. The parity plot in Fig. 4d compares the CO adsorption energies on selected Cu monolayer alloys obtained from the neural network model and DFT calculations. The inset in Fig. 4d depicts the geometry of the model system. By constructing a reliable machine learning model that enables high-throughput computation of key descriptors such as adsorption energy, d-band center, and coordination number, catalytic activity, optimal composition, and active sites can be predicted and understood for various potential materials and reaction pathways. Through the machine-learned chemisorption model, it was determined that Cu polymetallic compounds with a 100-capped structure exhibit lower overpotentials and higher selectivity for the electroreduction of CO_2_–C_2_ species. Figure 4e presents the relationship between each principal feature and the host metal M. It can be observed that, for all metal alloys, the d-band center can be adjusted through strain and ligand engineering due to its low sensitivity to different metals, while other factors show slight variations across the periodic table. This chemisorption model greatly facilitates the capture of complex nonlinear adsorbate–substrate interactions and deepens the understanding of chemical bonding on metal surfaces. Moreover, it opens up new avenues for the subsequent design of intricate metal-based catalysts. One constructive comment is that the authors have tested their model on a relatively small set of catalysts. While the results are promising, it would be interesting to assess the model's performance on a larger dataset of catalysts. Furthermore, it would be advantageous to compare the effectiveness of the chemisorption model augmented by machine learning with alternative methodologies that have been employed for catalyst screening in CO_2_ electroreduction.

#### Intermetallic Compounds

In the context of CO_2_ reduction reactions and the hydrogen uptake reaction (HER), the CO adsorption energy serves as a representative descriptor for predicting hydrocarbon production activity. This approach of using adsorption energy as a predictive tool can also be applied to general HER reactions. To identify potential selective catalysts for CO_2_ reduction and HER, Tran et al. developed a workflow based on surrogate optimization and active machine learning, which allowed for the screening of 1499 intermetallic compounds [69]. This study successfully identified 54 intermetallic surfaces with excellent CO_2_ reduction descriptors and 102 intermetallic surfaces with excellent HER descriptors. By combining machine learning techniques with DFT calculations, the feasibility of predicting the performance of electrocatalysts was demonstrated through the screening of alloys composed of 31 different elements. This integration of machine learning with DFT computations enables accelerated calculations and cost savings.

The specific workflow followed in this study involved initially using DFT calculations to verify the adsorption energies of various sites. The DFT results were then stored in a database for retraining the machine learning model, which was subsequently used for screening purposes. This iterative process formed a closed feedback loop, encompassing DFT verification, machine-learning-based screening, and retraining. To enable machine learning of catalyst descriptors, the researchers developed a fingerprint method for numerical representation of the adsorption sites in intermetallic compounds. Each element type was described using a four-number vector, incorporating the element's atomic number, Pauling electronegativity, atomic number coordination with the adsorbate, and the median adsorption energies between adsorbates and elements. An automated machine learning package called TPOT [70] was employed to select a suitable regression method for predicting adsorption energies. An iterative approach was utilized to calculate prediction errors, median absolute deviations over time, and the optimal number of surfaces over time. By analyzing all 19,644 sites computed by DFT, the researchers demonstrated that the performance of intermetallic compounds could be predicted based on the number and distribution of potential active sites. The researchers simulated the t-SNE plots of all adsorption sites using DFT and identified 258 different surfaces with low coverage ΔEH values. The intermetallic compounds screened using near-optimal ΔEH values were validated against relevant literature sources [70–73]. This work accelerates the theoretical discovery of CO catalysts and serves as an effective tool for screening candidate catalysts from a relatively large search space, guiding subsequent experimental studies. However, this design approach does not address other important aspects of catalyst screening, such as surface stability and catalyst cost. It is worth noting that this design approach can be extended to other reaction chemistries by employing appropriate thermodynamic descriptors.

### Optimization and Design

#### Gold/Silver Nanoparticles

**Fig. 5:**
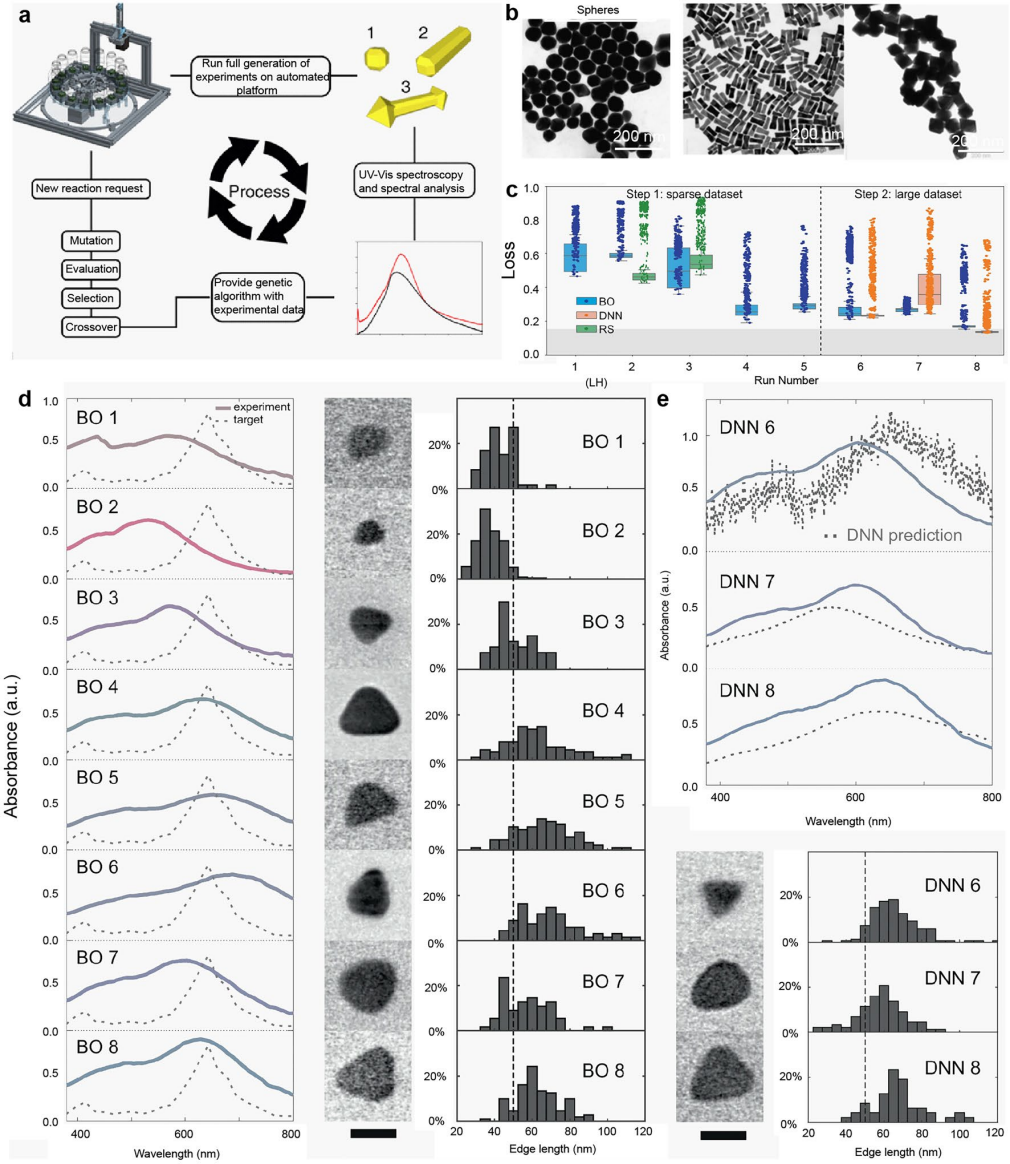
Machine-learning-based synthesis of gold and silver nanoparticles. **a** A schematic representation of a platform workflow depicting the hierarchical evolution of AuNPs. **b** TEM images showcasing gold nanospheres, gold nanorods, and gold nanooctahedrons. Adapted with permission from Ref. [46]. Copyright 2020, The Author(s). **c** Optimization of the synthesis process for silver nanoparticles. The absorbance spectra of the most proficient **d** BO and **e** DNN models, coupled with the concurrent size distribution analysis of triangular prisms in the solution, substantiate the efficacy of the selected loss function in facilitating the convergence of spectra toward the designated target spectrum and the triangular prisms toward an edge length of 65 nm. The scale bars in the images correspond to a length of 50 nm. Reproduced with permission from Ref. [47]. Copyright 2021, The Author(s)

Nanoparticles have a significant impact on various research fields, such as surface-enhanced Raman scattering, drug delivery, and biomolecular carriers. The unique characteristics of nanoparticles lie in the fact that their size, morphology, and surface chemistry profoundly influence their optical, electrical, and magnetic properties. Achieving precise control over particle size and morphology requires careful consideration of various experimental conditions, including reagent concentration ratio, reaction time, temperature, and external environment. This poses significant challenges in the controllable synthesis of nanoparticles. However, with the advancements in artificial intelligence, machine learning is increasingly being employed to expedite the development of controllable nanoparticles. Machine learning and optimization algorithms, such as Bayesian optimization (BO) and genetic algorithms, are now employed to achieve this precise control, thereby significantly reducing experimental time and resource consumption. For instance, in the preparation of gold nanoparticles, the acquisition of UV signals from known gold nanoparticles is essential to establish a spectral target for an automated system. Subsequently, a genetic algorithm is employed to synthesize gold nanoparticles based on the spectral target (Fig. 5a) [46]. This approach enables the easy generation of nanoparticles with diverse morphologies, as demonstrated by the transmission electron microscopy (TEM) images of gold nanospheres, gold nanorods, and gold nanooctahedrons prepared using the automated platform (Fig. 5b). In the case of silver nanoparticles, a microfluidic high-throughput experimental platform (THE) is utilized to obtain a large amount of experimental data with a minimal number of materials. BO is employed to guide the THE loop, as it efficiently explores the parameter space and targets specific material properties using a limited number of datasets [74, 75]. The two-step framework combines BO with deep neural networks (DNNs) to evaluate the optimization performance. In each iteration, the BO algorithm determines the experimental conditions for the subsequent cycle based on the trade-off between minimizing the loss and reducing the uncertainty determined by the loss function. As depicted in Fig. 5c [47], the median loss for the best BO performance decreases rapidly in the initial iterations, whereas random sampling (RS) shows an increasing trend. By introducing a DNN in the sixth run, lower median loss is achieved compared to BO in the eighth run. The absorbance peak of the BO samples converges rapidly toward the target value (645 nm) as the number of experiments increases. When BO and DNN are combined, the predicted spectrum becomes smoother in the seventh run after initially exhibiting some noise in the sixth run. A machine learning approach was employed to expedite the optimization of fabricated TiO_2_ nanotube micropatterns (TNM) and the doping of AgNPs [54] through electrochemical deposition. The findings of the study indicate an enhancement in the antibacterial performance with an increase in the diameter of the nanotubes [54]. Further details pertaining to these biomaterials will be elaborated in the subsequent section.

#### Hybrid Materials

**Fig. 6:**
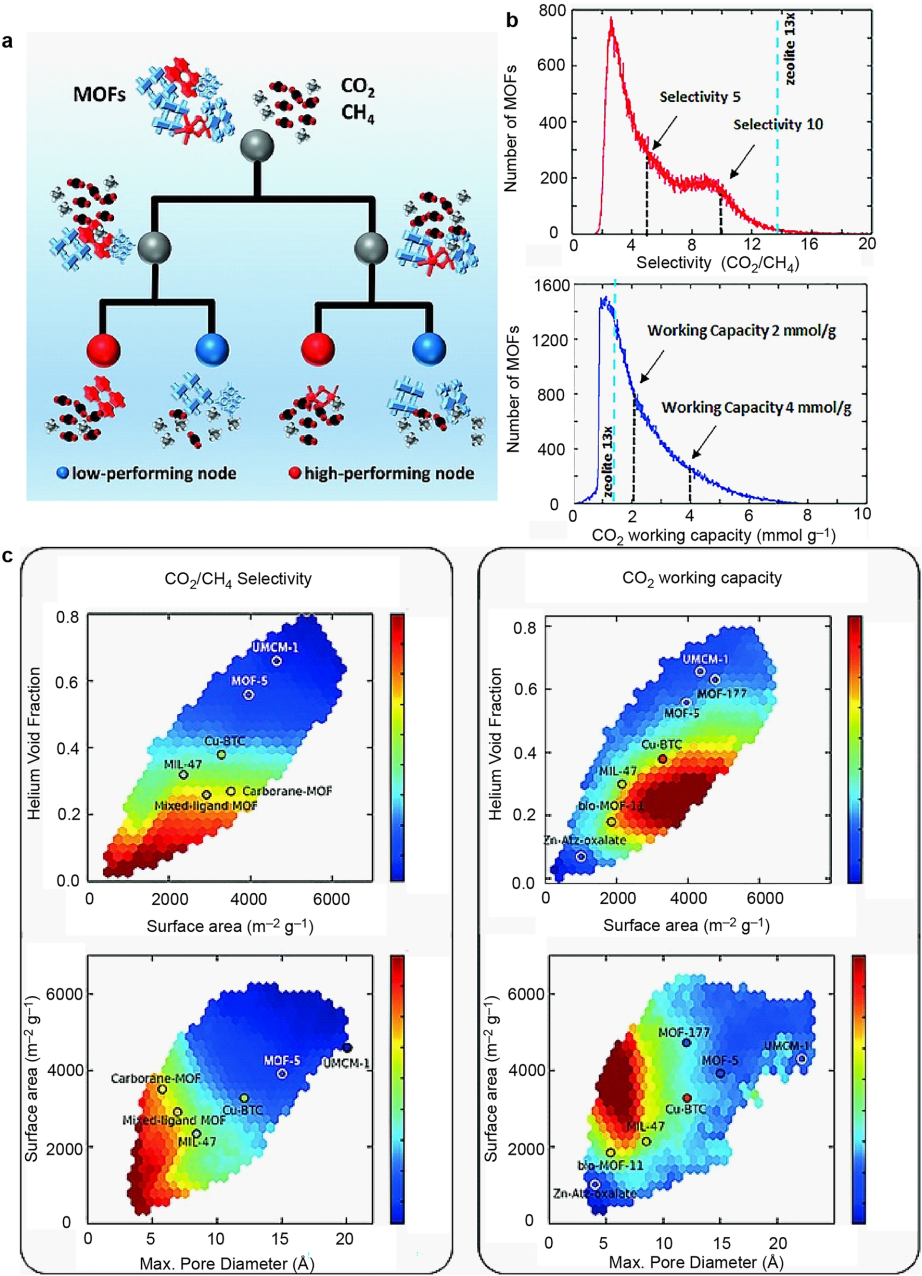
Identify high-performance metal–organic framework materials accurately through the utilization of machine-learning-based QSPR models. **a** Schematic representation of collaborative approach. **b** The quantity of screened molecules as a function of singlet–triplet splitting (ΔEST) and oscillator strength. **c** Linear model prediction based on data derived from TD-DFT, alongside neural network predictions on TD-DFT-derived data. Reproduced with permission from Ref. [51]. Copyright 2016 WILEY‐VCH Verlag GmbH & Co. KgaA, Weinheim

MOFs are porous materials composed of metal nodes and organic linkers, exhibiting exceptional porosity and a vast internal surface area. Their structure encompasses a wide range of organic and inorganic components, rendering them versatile for numerous applications, including gas storage, separation, catalysis [76], electrocatalysis [77], and biomedicine [78–80]. Despite extensive preparation and study of thousands of MOFs, their theoretical capacity to yield an infinite number of nanoporous materials renders it impossible to identify the best-performing MOFs solely through experimental means. However, by utilizing binary decision number (DT) and support vector machine (SVM) models calibrated with 325,000 MOF structures, high-performance MOFs can be accurately identified based on their binding properties, such as pore size, porosity, and surface area [51]. This approach achieves a remarkable recognition rate of up to 90% (Fig. 6a). The quantitative structure–property relationship (QSPR) model serves as an efficient computational tool for screening extensive structural libraries, thus facilitating the discovery of MOF sorbent materials for methane purification. The CO_2_ working capacity and CO_2_/CH_4_ selectivity distribution of the entire database are depicted in Fig. 6b, with approximately 60% of the databases exhibiting a CO_2_ working capacity exceeding 2 mmol g^−1^, and approximately 10% surpassing 4 mmol g^−1^. Regarding selectivity, around 40% of the databases showcase CO_2_/CH_4_ selectivity greater than 5, while 10% display selectivity surpassing 10. Figure 6c illustrates an interaction scatter plot of the maximum pore size, porosity, and surface area of 324,500 MOFs, with the CO_2_/CH_4_ selectivity and CO_2_ working capacity represented by color mapping. MOFs with lower surface area, pore size, and porosity exhibit selectivity values exceeding 5. In MOFs possessing a surface area below 1000 m^2^ g^−1^ and a porosity below 0.1, the CO_2_ working capacity decreases below 1 mmol g^−1^. The DT predictions are based on a simple binary rule, where a porosity below 0.32 and a pore size below 8.30 Å correspond to selectivity > 5 for CO_2_/CH_4_, while a porosity below 0.27 and a pore size below 6.6 Å correspond to selectivity > 10. Additionally, SVM was employed to screen extensive structural databases, aiming to reduce the computational demands of grand canonical Monte Carlo (GCMC) simulations. The relationship between the number of high-performing MOFs and various sensitivity thresholds indicates that by performing GCMC simulations on only 23% of the database, approximately 90% of the high-performance MOFs can be recovered. Consequently, the QSPR model emerges as an effective tool for predicting the CO_2_ working capacity and CO_2_/CH_4_ selectivity of MOFs, thereby facilitating their application in methane purification. The authors utilized a dataset comprising 79 MOFs and calculated various features encompassing geometric, topological, and chemical descriptors to train the QSPR model. The model's performance was evaluated using statistical metrics, and a feature selection analysis was conducted to identify the most influential features for predicting the properties of interest. The results demonstrated the QSPR model's accuracy in predicting the CO_2_ working capacity and CO_2_/CH_4_ selectivity of MOFs, with geometric descriptors such as surface area and pore volume identified as the most significant features for predicting these properties.

**Fig. 7:**
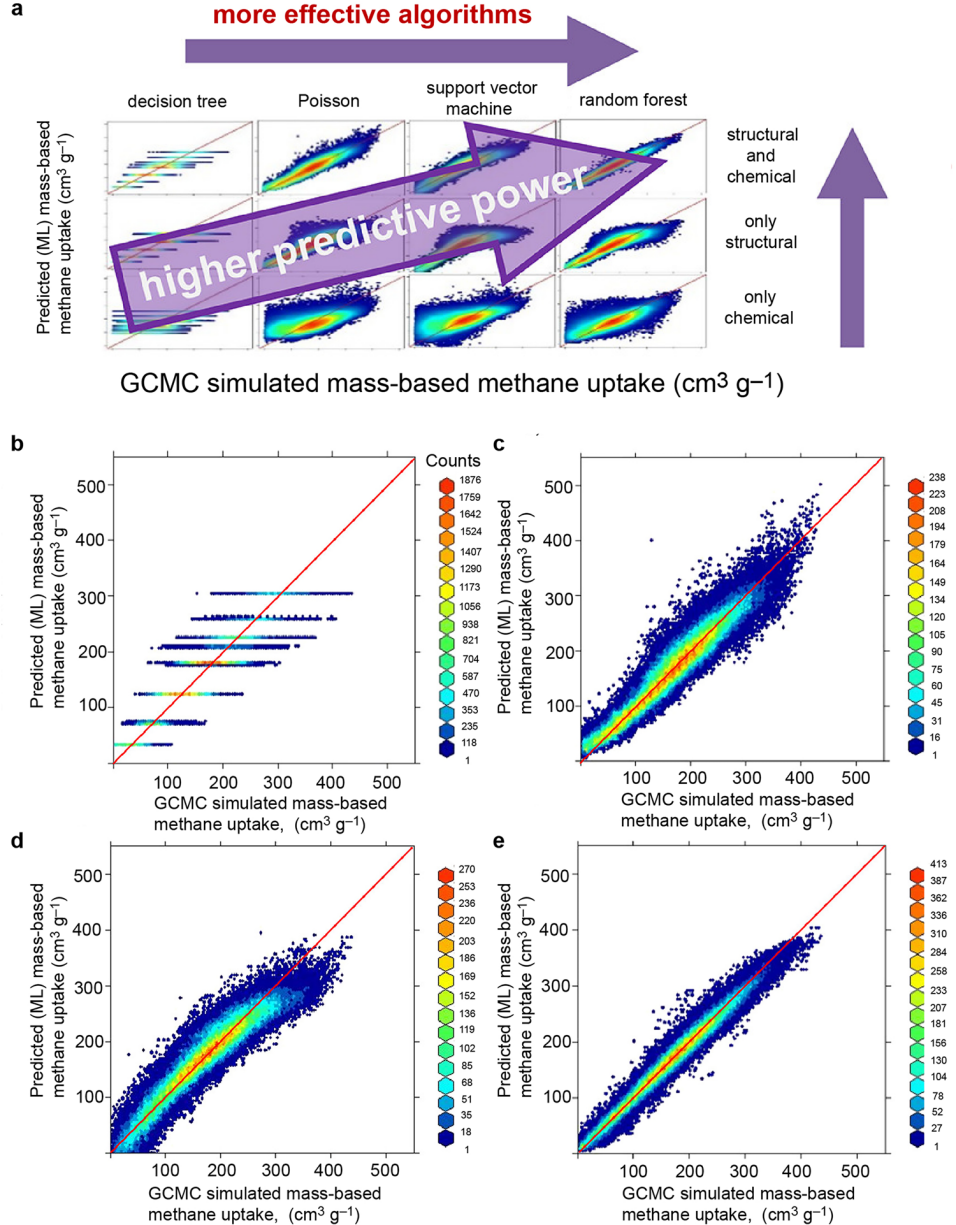
Evaluation of structural and chemical descriptors and various machine learning algorithms, including decision trees, Poisson regression, support vector machines, and random forests to predict methane uptake on metal–organic frameworks. **a** Structural descriptors and chemical descriptors were introduced for adsorption analysis. Parity plots illustrating the correlation between predicted mass-based methane uptake (cm^3^ g^−1^) derived from machine learning (ML) models and those obtained through Grand Canonical Monte Carlo (GCMC) simulations, incorporating structural and chemical variables for **b** DT, **c** Poisson, **d** SVM, and **e** RF models. The red diagonal line in each plot represents a 45° line, denoting perfect alignment between ML predictions and GCMC simulation outcomes. The color scale employed in the plots signifies the frequency of occurrences or the count of hypothetical metal–organic frameworks (hMOFs) exhibiting concordant GCMC and ML results. Reproduced with permission from Ref. [50]. Copyright 2017 Royal Society of Chemistry

As we are aware, natural gas is an abundant resource, with methane being its primary constituent. One notable advantage of methane as a fuel is its high combustion energy per unit of carbon dioxide compared to other hydrocarbons. However, its volumetric energy density under ambient conditions is merely 0.11% of that of gasoline. Therefore, finding suitable materials for methane adsorption becomes imperative. MOFs are widely recognized for their unique properties of regularity, diversity, and designability, which facilitate computer-aided screening [81, 82]. Nonetheless, traditional molecular simulations as screening tools are highly time-consuming due to the vast number of MOF structures involved. In response, considerable efforts have been devoted to employing machine learning in this field. Previous work has involved utilizing comprehensive databases of various adsorbent materials and conducting GCMC simulations to explore the relationship between structural properties and adsorption [83–92]. Regression models and radial distribution functions have been employed as predictors to estimate CO_2_ and N_2_ uptake [86]. Other studies have utilized structural variables to predict CH_4_ uptake, achieving an *R*^2^ value of 0.85 [87]. Classification methods based on quantitative structure–property relationship have been used to predict optimal MOFs for CO adsorption. Furthermore, structural properties such as surface area, crystal density, porosity, pore size, and heat of adsorption have been employed to predict CH_4_ uptake in MOFs. In addition to characterizing adsorption properties using structural features, comprehensive models have been developed to better elucidate the chemical interactions of MOFs. These models incorporate not only the physical properties of MOFs, such as surface organics, density, porosity, and crystal structure, but also introduce new variables, including the degree of unsaturation and electronegativity, as chemical predictors. Pardakhti et al. demonstrated that incorporating chemical variables into machine-learning-based material analysis can enhance prediction accuracy without sacrificing computational speed [50]. The DT algorithm, illustrated in Fig. 7a, employs if–then logistic rules to train the classification, while Poisson regression, a generalized linear model, utilizes regression directly associated with the model coefficients. The support vector machine method, a widely used classification technique, is adapted for nonlinear kernel applications. Figure 7b displays a comparative parity diagram that combines the physical structure and chemical characteristics of the materials. It is evident that the prediction accuracy significantly improves from the DT model to the random forest model (Fig. 7c). This study demonstrated that machine learning algorithms are relatively cost-effective compared to molecular simulations, and incorporating chemical variables into machine-learning-based material analysis can greatly enhance prediction accuracy and computational speed. In conclusion, the use of machine learning models for predicting methane adsorption performance of MOFs based on combined structural and chemical descriptors is a promising approach. The results of the study demonstrate the effectiveness of the developed model in predicting methane adsorption capacities of MOFs with high accuracy. The combination of structural and chemical descriptors provided a more comprehensive representation of MOFs, leading to better model performance. Furthermore, the proposed method has the potential to significantly accelerate the screening process for MOFs, which can save a considerable amount of time and resources in the development of new materials for gas storage and separation applications. However, there are some limitations that need to be addressed in future studies. First, the dataset used in this study is relatively small, which may limit the generalizability of the developed model. Future studies could benefit from a larger and more diverse dataset to improve the robustness of the model. Additionally, the proposed method only considers methane adsorption performance, and other gas adsorption properties could be included to provide a more comprehensive understanding of the adsorption behavior of MOFs.

### Process Simulation and Manufacturing

#### A Mobile Robotic Chemist

The synthesis of new materials is often accompanied by a wealth of data and intricate parameters, necessitating advanced methods to optimize manufacturing processes. Chemical synthesis routes for materials involve numerous potential transitions at each step, ranging from tens to thousands, which complicates the manufacturing process. Consequently, the consideration of highly complex systems and a vast array of potential transformations becomes essential. Within these combinations, various competing parameters such as time, cost, purity, and toxicity impact the manufacturing efficiency and product quality. Consequently, traditional experimental methods are no longer suitable for the synthesis and development of new materials. Organic chemists were among the first to recognize the immense potential of computer technology in chemical synthesis. Over 50 years ago, Corey's "Organic Analog Synthesis Program (OCSS)" attempted to automate the chemical synthesis of materials by harnessing the power of computers. This study demonstrated that, under specific conditions and synthetic rules, computers can effectively replace human experts and even surpass their efficiency [93]. Researchers at the University of Liverpool have developed an intelligent mobile robotic scientist capable of performing continuous, autonomous experiments over long periods of time [59]. It represents the first robotic scientist with the ability to independently decide which chemistry experiments to undertake next, leading to the discovery of a novel catalyst for hydrogen production from water. Equipped with a solid dispensing station, the robot can accurately measure solid components and transfer them into sample vials. Despite having a similar size to a human, the 400 kg robot possesses exceptional dexterity, enabling it to perform various tasks within the laboratory, such as vial handling, instrument operation, and intact sample storage. Unlike humans, the robot operates with unlimited patience and can work for up to 21.5 h per day, pausing solely for recharging. During an 8-day period, the robot executed 688 experiments, working a total of 172 h. It accomplished this through 319 movements and 6,500 operations, covering a distance of 2.17 km. The robot successfully identified the optimal catalyst formulation, a mixture of NaOH, L-cysteine, sodium disilicate, and P10, resulting in the highest hydrogen evolution rate (HER) of 21.05 µmol h^‒1^, a performance six times greater than that achieved under the starting conditions.

To investigate the algorithm's dependence on starting conditions, the researchers conducted 100 simulations with random initial conditions. They discovered that an experimental protocol achieving 95% HER performance could be identified after approximately 160 simulations. Although this AI-driven method is 1000 times faster than manual experimentation, it requires two years to establish such a platform. Further advancements are necessary to expedite the platform's construction and facilitate the development of artificial intelligence-powered materials science. Consequently, reducing the time and cost associated with building such a robotic platform is crucial to attract more laboratories and companies to utilize and promote its applications. While the extended working hours and lack of fatigue exhibited by the robotic scientist are advantageous, its ability to address unforeseen or complex experimental scenarios may be limited. To overcome these limitations, integrating machine learning algorithms, advanced perception systems, and fostering collaborations with human experts are potential avenues for enhancing the robot's adaptability and problem-solving capabilities. These advancements would contribute to a more comprehensive understanding of the robotic scientist's potential and facilitate its integration into materials science research and development.

#### Biomedicine

The design and fabrication of biomaterials are significant objectives in the field of biomedicine, where AI plays an increasing role in improving manufacturing processes. Conventional preparation methods are often laborious and time-consuming, necessitating the exploration of approaches that require fewer experiments and raw materials while achieving biomaterials with desirable properties [88–95]. Micropatterning techniques offer a means to miniaturize and integrate materials with distinct characteristics onto platforms, enabling efficient screening of biomaterials using minimal samples [53, 54, 96–98]. Among these techniques, bipolar electrochemistry has gained considerable popularity for constructing chemically or structurally gradient micropatterns [44, 99]. Given the widespread use of TiO_2_ nanotubes in biomedicine, sensing, and photocatalysis, the fabrication of TiO_2_ nanotube micropatterns (TNM) through bipolar electrochemical methods has been explored for high-throughput applications [54, 100]. It has been observed that increasing the applied voltage leads to an expansion in the diameter of the nanotubes, accompanied by accelerated current flow and potential cracking of the titanium foil during anodization. The regulation of parameters such as temperature, stirring speed, and electrolyte concentration is crucial to prevent the titanium foil from fracturing. However, the control of multiple parameters poses significant challenges in experimental design, necessitating the adoption of machine learning techniques to address these issues. A machine learning approach is employed to constrain the experimental boundary conditions for bipolar electrochemistry, thereby expediting the optimization of fabricated TNMs [54]. Through recurrent machine learning and experimental validation, the optimal experimental parameters for TNMs within a broad range of diameters can be determined with minimal experimentation. Notably, the predictions made by machine learning algorithms demonstrate superior agreement with experimental results compared to empirical predictions. Machine learning algorithms efficiently analyze electrochemical data, establish experimental boundaries, and optimize material fabrication. DT models are particularly suitable for classification tasks, enabling the identification of cutoff points and establishment of experimental boundary conditions. Gradient boosting regression tree (GBRT) models, on the other hand, learn data patterns and facilitate innovative design and manufacturing. The optimized TNMs can be further employed for high-throughput investigations in various fields, including biomedical devices, drug delivery, metal doping, photocurrent screening, corrosion resistance, photovoltaic cells, sensors, photoelectrochemical water splitting, and microfluidics. To illustrate, AgNPs were doped through electrochemical deposition while preserving the nanotube structure of TNM depicts the increase in antibacterial performance as the diameter of the nanotubes grows. Notably, we possess significant expertise in the biomedicine field [98–109].

Given the data-intensive nature of AI, the abundant data generated by microfluidics have provided fertile ground for the development of AI in this field. Machine learning techniques have been widely applied in various application areas within microfluidics. These include the optimization of biosensors [110, 111], cell detection and identification [99, 112, 113], disease diagnosis [114–116], drug discovery [117], drug susceptibility testing [118], protein identification [119], and stem cell proliferation [120]. ML not only enables analysis and measurement in microfluidics but also plays a role in microchip design. The applications of ML in microchips encompass fluid property measurement, soft sensors, flow cytometry, cytopathology, and glucose determination [121]. Furthermore, microchips can enhance the accuracy and speed of bioassays [119]. The integration of ML and microchips has demonstrated potential in image classification for tuberculosis detection [110]. In summary, the use of machine learning techniques in the design and fabrication of biomaterials, particularly in the context of bipolar electrochemistry and microfluidics, holds great promise. These techniques enable the optimization of experimental parameters, facilitate innovative design and manufacturing, and enhance the accuracy and speed of bioassays. The integration of machine learning with microchips offers new possibilities for applications such as disease diagnosis, treatment, and detection. Further research and development in these areas can lead to significant advancements in biomedicine.

## Conclusions and Outlook

The widespread availability of big data and continuous advancements in computer computing power have contributed to the rapid development of AI, particularly machine learning. AI technologies have experienced significant progress and have found application across various disciplines. AI methods, being driven by data, eliminate the need for constructing complex physical models or dealing with cumbersome empirical parameters. This paradigm shift has facilitated a transition from traditional causal exploration to the establishment of flexible relationships with the support of artificial intelligence. Furthermore, under specific conditions and synthesis rules, mobile robots have the potential to fully replace human experts, thereby alleviating the burden on human resources. Additionally, machine learning methods have successfully been employed in the preparation of nanomaterials such as bimetallic materials, carbon nanotubes, intermetallic compounds, MOFs, OLEDs, gold/silver nanoparticles, and biomaterials. These nanomaterials represent a highly active research field in materials science and have demonstrated excellent performance when synthesized using machine learning techniques.

Currently, the application of machine learning in the field of materials science is still in its nascent stage, and numerous challenges and issues need to be addressed. One major challenge is the scarcity of sufficient data. While AI has demonstrated remarkable potential, challenges remain. Data availability and standardization are critical for building robust machine learning models. Collaborative efforts to create shared databases with uniform protocols are essential for overcoming these limitations. Additionally, model interpretability remains a key focus, with explainable AI methods being developed to align predictions with physical principles. To address computational constraints, integrating AI with quantum computing offers a promising avenue. Future research should also emphasize integrating domain knowledge with machine learning algorithms to ensure interpretable and physically consistent results. To establish effective datasets, it is necessary to ensure uniform standards for the data, including using the same experimental system, experimental conditions, and dimensional variables. Data in material science suffer from high acquisition costs and lack of standardized processing. Despite researchers in the field sharing common research directions, experimental conditions often vary significantly. Attempting to unify data obtained under different experimental conditions can lead to overfitting. Simulated data cannot fully capture the specific experimental conditions, and certain characteristic parameters in experimental conditions are difficult to obtain comprehensively. Machine learning heavily relies on robust data support, as without adequate data, it cannot reflect the true value of the learning process. Moreover, experimentally obtained data in materials science exhibit complex and chaotic features, making it challenging for machine learning techniques to rapidly identify, classify, and establish associations. Consequently, extracting the crucial features from original data becomes arduous for machine learning algorithms. Recent advancements in explainable AI (XAI) techniques are being leveraged to tackle these challenges, enabling researchers to align ML predictions with established physical and chemical principles. Furthermore, emerging trends such as hybrid AI models that combine physics-based simulations with ML have shown promise in addressing data scarcity and improving interpretability. Additionally, the existing practical algorithms and models often lack generality and are limited to specific scenarios. The discovery of new materials is frequently accompanied by the development of new preparation conditions and rules. Furthermore, scientists working in the materials field typically possess scientific backgrounds in materials and physics, lacking familiarity with computer-based machine learning. This knowledge gap hinders the progress of machine learning in materials innovation. Another aspect to consider is whether machine learning can entirely replace traditional experimental research, despite its potential in the development and application of novel materials. Further investigation is necessary to determine whether the implicit correlations and rules derived from machine learning align with the intrinsic characteristics of the materials. Verifying this alignment requires substantial effort.

Finally, we present our perspectives on the challenges and opportunities in this field.

1. One of the primary challenges is the construction and management of databases. Effectively storing, managing, and analyzing vast amounts of data presents a complex problem. As the concept of material genome gains traction, accurately and comprehensively characterizing the relationships between composition, structure, and properties of materials becomes crucial for the research and development of new materials. Establishing more comprehensive material databases necessitates collaborative efforts among scientific researchers worldwide, along with the creation of material library collections. This involves establishing material-specific data sets and transforming the material databases into AI-readable digital formats.
2. The integration of machine learning and high-throughput computing offers transformative potential for sustainability by optimizing resource use and reducing experimental redundancies. Both approaches are adept at extracting valuable insights from large data sets. High-throughput computing functions as an efficient computational tool, lacking independent learning capabilities. In contrast, machine learning possesses the ability to learn autonomously. By combining the strengths of both approaches, the efficiency of new material screening can be further enhanced. This integration leverages standardized technical parameters and the large volume of high-throughput computing alongside the self-learning ability of machine learning.
3. There is room for improvement in machine learning algorithms. Uncertainty in machine learning arises from three major aspects. Firstly, the uncertainty of input data encompasses geometry uncertainty, model parameter uncertainty, boundary condition uncertainty, and initial condition uncertainty. Secondly, the uncertainty of model form includes model bias and limited computational budgets. Thirdly, the uncertainty of numerical methods encompasses discretization error, iterative error, round-off error, and coding error. Current research focuses on quantitatively analyzing data uncertainty, understanding the uncertainty of machine learning models themselves, and comprehending the learning process of deep learning networks and the resulting prediction functions. These areas represent the forefront of current research interests.
4. Feature engineering is crucial in machine learning, wherein it involves the careful selection, extraction, and transformation of descriptors into a suitable format for machine learning algorithms. This approach has emerged as a powerful tool in materials science, facilitating the efficient selection of relevant features for specific problems. Moreover, descriptor-based feature engineering provides insights into the underlying physical and chemical processes governing material properties. Descriptors can be categorized into two main types: structural descriptors and compositional descriptors. Structural descriptors capture the geometry and topology of materials, including bond angles, bond lengths, coordination numbers, and surface area. They offer valuable information about the arrangement of atoms within a material, enabling predictions of mechanical, electronic, and optical properties. Compositional descriptors, on the other hand, describe the elemental composition of a material, such as the atomic fractions of constituent elements and their distribution throughout the material. Compositional descriptors provide insights into the chemical makeup of a material and can predict its chemical, thermal, and magnetic properties [122]. Utilizing descriptors, researchers have successfully predicted various material properties, such as crystal structure, bandgap, melting point, and elastic modulus [123]. For instance, machine learning models trained on structural and compositional descriptors have achieved high accuracy in predicting the elastic modulus of materials, even for materials that have not been experimentally tested [123]. Despite the numerous advantages of descriptor-based feature engineering, several challenges must be addressed. One key challenge is the selection of relevant descriptors from the vast array of available options in materials science. The presence of highly correlated descriptors can lead to overfitting of models [16]. The challenge of selecting relevant descriptors from the vast array available in materials science can be addressed through a systematic approach combining domain knowledge, computational methods, and data-driven strategies. Initially, leveraging domain expertise and insights from literature or experimental studies can guide the prioritization of descriptors with known physical significance. Statistical and machine learning techniques, such as filter methods, wrapper methods, or embedded methods (e.g., Lasso regression or tree-based models), can be employed to identify the most relevant features. Dimensionality reduction methods, including principal component analysis (PCA) and t-SNE, can further aid in isolating key descriptors by reducing redundancy. Advanced feature engineering techniques, such as composite descriptor creation and automated feature generation, enhance the representation of material properties. Additionally, high-throughput screening and explainable AI methods, like Shapley values, can be used to evaluate and rank descriptors based on their predictive contributions. Validation through cross-validation, model performance metrics, and experimental feedback ensures the robustness of selected descriptors. Finally, accessing established databases, developing descriptor ontologies, and utilizing active learning approaches provide valuable frameworks for refining and optimizing feature selection processes. Together, these strategies address the complexity of descriptor selection, facilitating the development of predictive and interpretable models in materials science. Another challenge lies in the interpretability of machine learning models based on descriptors. While descriptors themselves are interpretable, complex machine learning models, especially those employing deep learning algorithms, can be difficult to interpret. This lack of interpretability poses difficulties in understanding the underlying physical and chemical processes governing material properties [124]. Standardization of data formats and reporting protocols for materials data is also a major challenge. Without standardized formats, sharing, comparing, and interpreting data across different research groups and databases become cumbersome. The challenge of data preprocessing and standardization in materials science can be addressed through the adoption of unified data formats, the establishment of robust reporting protocols, and the integration of computational tools designed for interoperability. Standardization initiatives, such as the Materials Project's Materials API and the Materials Data Facility's data repository aim to address this challenge by providing standardized formats and protocols, facilitating the sharing and integration of materials data for machine learning and AI applications [125]. Automated preprocessing pipelines, incorporating techniques for data cleaning, normalization, and transformation, streamline the preparation of materials datasets for machine learning applications. Furthermore, the use of ontologies and metadata standards, such as the Chemical Markup Language (CML), facilitates semantic interoperability, allowing for more efficient data integration and interpretation. It is important to note that descriptors are not a substitute for domain expertise in materials science. While descriptors offer insights into material properties, they represent just one tool among many in the materials scientist's toolkit. Domain expertise remains essential for comprehending the complexities of materials science and designing materials with specific properties. Future research in this field holds promise for the development of novel descriptors, the incorporation of domain knowledge, the utilization of multiple descriptors, the enhancement of data quality and availability, and the creation of more interpretable machine learning models [88].
5. The integration of AI into materials science holds transformative potential for advancing sustainable development [126, 127]. By accelerating the discovery, design, and optimization of materials, AI can address global challenges such as resource efficiency, energy sustainability, and environmental impact reduction. Predictive modeling and optimization algorithms enable the identification of environmentally friendly alternatives to critical raw materials and the minimization of material waste, thereby promoting circular material flows and reducing dependence on non-renewable resources. Furthermore, AI facilitates the development of materials for renewable energy technologies, such as high-capacity battery components and green hydrogen catalysts, while enhancing their efficiency and longevity. Life-cycle assessment (LCA) frameworks, augmented by AI, allow researchers to quantify and mitigate the environmental impacts of materials throughout their production, use, and disposal stages. Predictive maintenance further extends material lifespans, contributing to resource conservation.

We firmly believe that the application of machine learning in materials science is just at the beginning of its journey. We anticipate that artificial intelligence will find increasingly widespread use in materials research and development. As artificial intelligence continues to advance, we expect a steady growth in the development and practical application of new materials, driven by the synergistic capabilities of machine learning and materials science. Emerging trends, such as AI-augmented robotics for autonomous material synthesis and in situ experimentation, further illustrate the transformative impact of AI. Future research should also prioritize ethical considerations, sustainability, and the integration of domain-specific knowledge to ensure that AI applications in materials science align with broader societal goals.

## References

[1] L. Liu, M. Bi, Y. Wang, J. Liu, X. Jiang et al., Artificial intelligence-powered microfluidics for nanomedicine and materials synthesis. Nanoscale **13**, 19352–19366 (2021). 10.1039/D1NR06195J 34812823 10.1039/d1nr06195j

[2] Z. Li, S. Wang, Xin Toward artificial intelligence in catalysis. Nat. Catal. **1**, 641–642 (2018). 10.1038/s41929-018-0150-1

[3] R. Batra, L. Song, R. Ramprasad, Emerging materials intelligence ecosystems propelled by machine learning. Nat. Rev. Mater. **6**, 655–678 (2020). 10.1038/s41578-020-00255-y

[4] K. Honkala, A. Hellman, I.N. Remediakis, A. Logadottir, A. Carlsson et al., Ammonia synthesis from first-principles calculations. Science **307**, 555–558 (2005). 10.1126/science.1106435 15681379 10.1126/science.1106435

[5] R.M. Wentzcovitch, J.L. Martins, G.D. Price, Ab initio molecular dynamics with variable cell shape: application to MgSiO_3_. Phys. Rev. Lett. **70**, 3947–3950 (1993). 10.1103/PhysRevLett.70.3947 10054006 10.1103/PhysRevLett.70.3947

[6] V. Oliveira, R. Vilar, Finite element simulation of pulsed laser ablation of titanium carbide. Appl. Surf. Sci. **253**, 7810–7814 (2007). 10.1016/j.apsusc.2007.02.101

[7] Z. Wang, H. Zhang, J. Ren, X. Lin, T. Han et al., Predicting adsorption ability of adsorbents at arbitrary sites for pollutants using deep transfer learning. npj Comput. Mater. **7**, 1–9 (2021). 10.1038/s41524-021-00494-9

[8] Y. Han, I. Ali, Z. Wang, J. Cai, S. Wu et al., Machine learning accelerates quantum mechanics predictions of molecular crystals. Phys. Rep. **934**, 1–71 (2021). 10.1016/j.physrep.2021.08.002

[9] Z. Wang, Y. Han, X. Lin, J. Cai, S. Wu et al., An ensemble learning platform for the large-scale exploration of new double perovskites. ACS Appl. Mater. Interfaces **14**, 717–725 (2022). 10.1021/acsami.1c18477 34967594 10.1021/acsami.1c18477

[10] K.T. Butler, D.W. Davies, H. Cartwright, O. Isayev, Walsh machine learning for molecular and materials science. Nature **559**, 547–555 (2018). 10.1038/s41586-018-0337-2 30046072 10.1038/s41586-018-0337-2

[11] J.J. Irwin, T. Sterling, M.M. Mysinger, E.S. Bolstad, R.G. Coleman, ZINC: a free tool to discover chemistry for biology. J. Chem. Inf. Model. **52**, 1757–1768 (2012). 10.1021/ci3001277 22587354 10.1021/ci3001277 PMC3402020

[12] A. Gaulton, L.J. Bellis, A.P. Bento, J. Chambers, M. Davies et al., ChEMBL: a large-scale bioactivity database for drug discovery. Nucleic Acids Res. **40**, D1100–D1107 (2012). 10.1093/nar/gkr777 21948594 10.1093/nar/gkr777 PMC3245175

[13] L.C. Blum, J.-L. Reymond, 970 million druglike small molecules for virtual screening in the chemical universe database GDB-13. J. Am. Chem. Soc. **131**, 8732–8733 (2009). 10.1021/ja902302h 19505099 10.1021/ja902302h

[14] L. Ruddigkeit, R. van Deursen, L.C. Blum, J.L. Reymond, Enumeration of 166 billion organic small molecules in the chemical universe database GDB-17. J. Chem. Inf. Model. **52**, 2864–2875 (2012). 10.1021/ci300415d 23088335 10.1021/ci300415d

[15] A. Belsky, M. Hellenbrandt, V.L. Karen, P. Luksch, New developments in the inorganic crystal structure database (ICSD): accessibility in support of materials research and design. Acta Crystallogr. B **58**, 364–369 (2002). 10.1107/s0108768102006948 12037357 10.1107/s0108768102006948

[16] S. Kirklin, J.E. Saal, B. Meredig, A. Thompson, J.W. Doak et al., The open quantum materials database (OQMD): assessing the accuracy of DFT formation energies. npj Comput. Mater. **1**, 15010 (2015). 10.1038/npjcompumats.2015.10

[17] E.O. Pyzer-Knapp, K. Li, A. Aspuru-Guzik, Learning from the Harvard clean energy project: the use of neural networks to accelerate materials discovery. Adv. Funct. Mater. **25**, 6495–6502 (2015). 10.1002/adfm.201501919

[18] Y.G. Chung, E. Haldoupis, B.J. Bucior, M. Haranczyk, S. Lee et al., Advances, updates, and analytics for the computation-ready, experimental metal–organic framework database: core MOF 2019. J. Chem. Eng. Data **64**, 5985–5998 (2019). 10.1021/acs.jced.9b00835

[19] F.H. Allen, R. Taylor, Research applications of the Cambridge structural database (CSD). Chem. Soc. Rev. **33**, 463 (2004). 10.1039/b309040j 15480471 10.1039/b309040j

[20] I.E. Castelli, D.D. Landis, K.S. Thygesen, S. Dahl, I. Chorkendorff et al., New cubic perovskites for one- and two-photonwater splitting using the computational materials repository. Energy Environ. Sci. **5**, 9034–9043 (2012). 10.1039/C2EE22341D

[21] Y. Wang, J. Xiao, T.O. Suzek, J. Zhang, J. Wang et al., PubChem: a public information system for analyzing bioactivities of small molecules. Nucleic Acids Res. **37**, W623–W633 (2009). 10.1093/nar/gkp456 19498078 10.1093/nar/gkp456 PMC2703903

[22] Z. Wang, Y. Han, J. Cai, A. Chen, J. Li, Vision for energy material design: a roadmap for integrated data-driven modeling. J. Energy Chem. **71**, 56–62 (2022). 10.1016/j.jechem.2022.03.052

[23] M.N. Gjerding, A. Taghizadeh, A. Rasmussen, S. Ali, F. Bertoldo et al., Recent progress of the computational 2D materials database (C2DB). 2D Mater. **8**, 044002 (2021). 10.1088/2053-1583/ac1059

[24] J.R. Quinlan, *C4.5: Programs for Machine Learning* (Elsevier, 2014)

[25] S. Chen, G.I. Webb, L. Liu, X. Ma, A novel selective naïve Bayes algorithm. Knowl. Based Syst. **192**, 105361 (2020). 10.1016/j.knosys.2019.105361

[26] V. Solovev, A. Tsivadze, G. Marcou, A. Varnek, Classification of metal binders by Naïve Bayes classifier on the base of molecular fragment descriptors and ensemble modeling. Mol. Inform. **38**, 1900002 (2019). 10.1002/minf.201900002 10.1002/minf.201900002 30969483

[27] V. Cherkassky, Y. Ma, Practical selection of SVM parameters and noise estimation for SVM regression. Neural Netw. **17**, 113–126 (2004). 10.1016/S0893-6080(03)00169-2 14690712 10.1016/S0893-6080(03)00169-2

[28] S.H. Lee, J. Mazumder, J. Park, S. Kim, Ranked feature-based laser material processing monitoring and defect diagnosis using k-NN and SVM. J. Manuf. Process. **55**, 307–316 (2020). 10.1016/j.jmapro.2020.04.015

[29] S. Yagiz, E. Ghasemi, A.C. Adoko, Prediction of rock brittleness using genetic algorithm and particle swarm optimization techniques. Geotech. Geol. Eng. **36**, 3767–3777 (2018). 10.1007/s10706-018-0570-3

[30] T. Cover, P. Hart, Nearest neighbor pattern classification. IEEE Trans. Inf. Theory **13**, 21–27 (1967). 10.1109/TIT.1967.1053964

[31] T. Adithiyaa, D. Chandramohan, T. Sathish, Optimal prediction of process parameters by GWO-KNN in stirring-squeeze casting of AA2219 reinforced metal matrix composites. Mater. Today Proc. **21**, 1000–1007 (2020). 10.1016/j.matpr.2019.10.051

[32] T. Sathish, S. Rangarajan, A. Muthuram, R.P. Kumar, Analysis and modelling of dissimilar materials welding based on K-nearest neighbour predictor. Mater. Today Proc. **21**, 108–112 (2020). 10.1016/j.matpr.2019.05.371

[33] R.E. Schapire, Explaining AdaBoost, in *Empirical Inference: Festschrift in Honor of Vladimir N. Vapnik*. ed. by B. Schölkopf, Z. Luo, V. Vovk (Springer, Berlin, 2013), pp.37–52. 10.1007/978-3-642-41136-6_5

[34] J. Li, C. Zhang, X. Zhang, H. He, W. Liu et al., Temperature compensation of piezo-resistive pressure sensor utilizing ensemble AMPSO-SVR based on improved Adaboost.RT. IEEE Access **8**, 12413–12425 (2020). 10.1109/ACCESS.2020.2965150

[35] W.-Y. Loh, Classification and regression trees. Wires Data Min. Knowl. Discov. **1**, 14–23 (2011). 10.1002/widm.8

[36] S. Kadali, S.M. Naushad, A. Radha-Rama-Devi, V.L. Bodiga, Biochemical, machine learning and molecular approaches for the differential diagnosis of Mucopolysaccharidoses. Mol. Cell. Biochem. **458**, 27–37 (2019). 10.1007/s11010-019-03527-6 30903511 10.1007/s11010-019-03527-6

[37] T. Masuda, T. Nishio, J. Kataoka, M. Arimoto, A. Sano et al., ML-EM algorithm for dose estimation using PET in proton therapy. Phys. Med. Biol. **64**, 175011 (2019). 10.1088/1361-6560/ab3276 31307027 10.1088/1361-6560/ab3276

[38] N.A. Karakatsanis, E. Fokou, C. Tsoumpas, Dosage optimization in positron emission tomography: state-of-the-art methods and future prospects. Am. J. Nucl. Med. Mol. Imaging **5**, 527–547 (2015). 10.1370/afm.825 26550543 PMC4620179

[39] A. Likas, N. Vlassis, J.J. Verbeek, The global k-means clustering algorithm. Pattern Recognit. **36**, 451–461 (2003). 10.1016/S0031-3203(02)00060-2

[40] Y. Liu, J. Wu, Z. Wang, X.-G. Lu, M. Avdeev et al., Predicting creep rupture life of Ni-based single crystal superalloys using divide-and-conquer approach based machine learning. Acta Mater. **195**, 454–467 (2020). 10.1016/j.actamat.2020.05.001

[41] T. Ueno, T.D. Rhone, Z. Hou, T. Mizoguchi, K. Tsuda, COMBO: an efficient Bayesian optimization library for materials science. Mater. Discov. **4**, 18–21 (2016). 10.1016/j.md.2016.04.001

[42] E. Gossett, C. Toher, C. Oses, O. Isayev, F. Legrain et al., AFLOW-ML: a RESTful API for machine-learning predictions of materials properties. Comput. Mater. Sci. **152**, 134–145 (2018). 10.1016/j.commatsci.2018.03.075

[43] X. Wang, P. Xie, B. Chen, X. Zhang, Chip-based high-dimensional optical neural network. Nano-Micro Lett. **14**, 221 (2022). 10.1007/s40820-022-00957-8 10.1007/s40820-022-00957-8 PMC9663775 36374430

[44] P. Nikolaev, D. Hooper, F. Webber, R. Rao, K. Decker et al., Autonomy in materials research: a case study in carbon nanotube growth. npj Comput. Mater. **2**, 16031 (2016). 10.1038/npjcompumats.2016.31

[45] R. Gómez-Bombarelli, J. Aguilera-Iparraguirre, T.D. Hirzel, D. Duvenaud, D. Maclaurin et al., Design of efficient molecular organic light-emitting diodes by a high-throughput virtual screening and experimental approach. Nat. Mater. **15**, 1120–1127 (2016). 10.1038/nmat4717 27500805 10.1038/nmat4717

[46] D. Salley, G. Keenan, J. Grizou, A. Sharma, S. Martín et al., A nanomaterials discovery robot for the Darwinian evolution of shape programmable gold nanoparticles. Nat. Commun. **11**, 2771 (2020). 10.1038/s41467-020-16501-4 32488034 10.1038/s41467-020-16501-4 PMC7265452

[47] F. Mekki-Berrada, Z. Ren, T. Huang, W.K. Wong, F. Zheng et al., Two-step machine learning enables optimized nanoparticle synthesis. npj Comput. Mater. **7**, 55 (2021). 10.1038/s41524-021-00520-w

[48] M. Sajjan, S.H. Sureshbabu, S. Kais, Quantum machine-learning for eigenstate filtration in two-dimensional materials. J. Am. Chem. Soc. **143**, 18426–18445 (2021). 10.1021/jacs.1c06246 34705449 10.1021/jacs.1c06246

[49] G.H. Gu, J. Noh, I. Kim, Y. Jung, Machine learning for renewable energy materials. J. Mater. Chem. A **7**, 17096–17117 (2019). 10.1039/c9ta02356a

[50] M. Pardakhti, E. Moharreri, D. Wanik, S.L. Suib, R. Srivastava, Machine learning using combined structural and chemical descriptors for prediction of methane adsorption performance of metal organic frameworks (MOFs). ACS Comb. Sci. **19**, 640–645 (2017). 10.1021/acscombsci.7b00056 28800219 10.1021/acscombsci.7b00056

[51] M.Z. Aghaji, M. Fernandez, P.G. Boyd, T.D. Daff, T.K. Woo, Quantitative structure–property relationship models for recognizing metal organic frameworks (MOFs) with high CO_2_ working capacity and CO_2_/CH_4_ selectivity for methane purification. Eur. J. Inorg. Chem. **2016**, 4505–4511 (2016). 10.1002/ejic.201600365

[52] M.-H. Lee, Machine learning for understanding the relationship between the charge transport mobility and electronic energy levels for n-type organic field-effect transistors. Adv. Electron. Mater. **5**, 1900573 (2019). 10.1002/aelm.201900573

[53] F. Li, Y. Li, K.S. Novoselov, et al. Bioresource upgrade for sustainable energy, environment, and biomedicine. Nano-Micro Lett. **15**, 35 (2023). 10.1007/s40820-022-00993-4 10.1007/s40820-022-00993-4 PMC9833044 36629933

[54] Y. Wang, G. Zheng, N. Jiang, et al. Nature-inspired micropatterns. Nat. Rev. Method. Prim. **3**, 68 (2023). 10.1038/s43586-023-00251-w

[55] A. Criminisi, Machine learning for medical images analysis. Med. Image Anal. **33**, 91–93 (2016). 10.1016/j.media.2016.06.002 27374127 10.1016/j.media.2016.06.002

[56] H.-C. Shin, H.R. Roth, M. Gao, L. Lu, Z. Xu et al., Deep convolutional neural networks for computer-aided detection: CNN architectures, dataset characteristics and transfer learning. IEEE Trans. Med. Imag. **35**, 1285–1298 (2016). 10.1109/TMI.2016.2528162 10.1109/TMI.2016.2528162 PMC4890616 26886976

[57] K.K. Yang, Z. Wu, F.H. Arnold, Machine-learning-guided directed evolution for protein engineering. Nat. Meth. **16**, 687–694 (2019). 10.1038/s41592-019-0496-6 10.1038/s41592-019-0496-6 31308553

[58] S. Ekins, A.C. Puhl, K.M. Zorn, T.R. Lane, D.P. Russo et al., Exploiting machine learning for end-to-end drug discovery and development. Nat. Mater. **18**, 435–441 (2019). 10.1038/s41563-019-0338-z 31000803 10.1038/s41563-019-0338-z PMC6594828

[59] B. Burger, P.M. Maffettone, V.V. Gusev, C.M. Aitchison, Y. Bai et al., A mobile robotic chemist. Nature **583**, 237–241 (2020). 10.1038/s41586-020-2442-2 32641813 10.1038/s41586-020-2442-2

[60] X. Ma, Z. Li, L.E.K. Achenie, H. Xin, Machine-learning-augmented chemisorption model for CO_2_ electroreduction catalyst screening. J. Phys. Chem. Lett. **6**, 3528–3533 (2015). 10.1021/acs.jpclett.5b01660 26722718 10.1021/acs.jpclett.5b01660

[61] M.F.L. De Volder, S.H. Tawfick, R.H. Baughman, A.J. Hart, Carbon nanotubes: present and future commercial applications. Science **339**, 535–539 (2013). 10.1126/science.1222453 23372006 10.1126/science.1222453

[62] X. He, D.J. Singh, P. Boon-On, M.-W. Lee, L. Zhang, Dielectric behavior as a screen in rational searches for electronic materials: metal pnictide sulfosalts. J. Am. Chem. Soc. **140**, 18058–18065 (2018). 10.1021/jacs.8b10685 30516996 10.1021/jacs.8b10685

[63] J. Wu, H. Yang, Platinum-based oxygen reduction electrocatalysts. Acc. Chem. Res. **46**, 1848–1857 (2013). 10.1021/ar300359w 23808919 10.1021/ar300359w

[64] Y. Nie, L. Li, Z. Wei, Recent advancements in Pt and Pt-free catalysts for oxygen reduction reaction. Chem. Soc. Rev. **44**, 2168–2201 (2015). 10.1039/C4CS00484A 25652755 10.1039/c4cs00484a

[65] A.S. Bandarenka, M.T.M. Koper, Structural and electronic effects in heterogeneous electrocatalysis: toward a rational design of electrocatalysts. J. Catal. **308**, 11–24 (2013). 10.1016/j.jcat.2013.05.006

[66] C.T. Campbell, Bimetallic surface chemistry. Annu. Rev. Phys. Chem. **41**, 775–837 (1990). 10.1146/annurev.pc.41.100190.004015

[67] W. Yu, M.D. Porosoff, J.G. Chen, Review of Pt-based bimetallic catalysis: from model surfaces to supported catalysts. Chem. Rev. **112**, 5780–5817 (2012). 10.1021/cr300096b 22920037 10.1021/cr300096b

[68] W.J.O.-T, Electronic structure and the properties of solids: the physics of the chemical bond. J. Mol. Struct. **71**, 355 (1981). 10.1016/0022-2860(81)85136-8

[69] K. Tran, Z.W. Ulissi, Active learning across intermetallics to guide discovery of electrocatalysts for CO_2_ reduction and H_2_ evolution. Nat. Catal. **1**, 696–703 (2018). 10.1038/s41929-018-0142-1

[70] R.S. Olson, R.J. Urbanowicz, P.C. Andrews, N.A. Lavender, L.C. Kidd, J.H. Moore, Automating biomedical data science through tree-based pipeline optimization, in *Applications of Evolutionary Computation*. ed. by G. Squillero, P. Burelli (Springer, Cham, 2016), pp.123–137. 10.1007/978-3-319-31204-0_9

[71] P.V. Cherepanov, M. Ashokkumar, D.V. Andreeva, Ultrasound assisted formation of Al-Ni electrocatalyst for hydrogen evolution. Ultrason. Sonochem. **23**, 142–147 (2015). 10.1016/j.ultsonch.2014.10.012 25453211 10.1016/j.ultsonch.2014.10.012

[72] M. Yamauchi, R. Abe, T. Tsukuda, K. Kato, M. Takata, Highly selective ammonia synthesis from nitrate with photocatalytically generated hydrogen on CuPd/TiO_2_. J. Am. Chem. Soc. **133**, 1150–1152 (2011). 10.1021/ja106285p 21204553 10.1021/ja106285p

[73] H. Liao, C. Wei, J. Wang, A. Fisher, T. Sritharan et al., A multisite strategy for enhancing the hydrogen evolution reaction on a nano-Pd surface in alkaline media. Adv. Energy Mater. **7**, 1701129 (2017). 10.1002/aenm.201701129

[74] M. Yamawaki, M. Ohnishi, S. Ju, J. Shiomi, Multifunctional structural design of graphene thermoelectrics by Bayesian optimization. Sci. Adv. **4**, eaar4192 (2018). 10.1126/sciadv.aar4192 29922713 10.1126/sciadv.aar4192 PMC6003749

[75] B. Yuan, G.M. Guss, A.C. Wilson, S.P. Hau-Riege, P.J. DePond et al., Machine-learning-based monitoring of laser powder bed fusion. Adv. Mater. Technol. **3**, 1800136 (2018). 10.1002/admt.201800136

[76] J. Wu, F. Xu, S. Li, P. Ma, X. Zhang et al., Porous polymers as multifunctional material platforms toward task-specific applications. Adv. Mater. **31**, e1802922 (2019). 10.1002/adma.201802922 30345562 10.1002/adma.201802922

[77] J. Meng, Z. Liu, X. Liu, W. Yang, L. Wang et al., Scalable fabrication and active site identification of MOF shell-derived nitrogen-doped carbon hollow frameworks for oxygen reduction. J. Mater. Sci. Technol. **66**, 186–192 (2021). 10.1016/j.jmst.2020.07.007

[78] J. Li, S. Song, J. Meng, L. Tan, X. Liu et al., 2D MOF periodontitis photodynamic ion therapy. J. Am. Chem. Soc. **143**, 15427–15439 (2021). 10.1021/jacs.1c07875 34516125 10.1021/jacs.1c07875

[79] J. Yang, X. Zhang, C. Liu, Z. Wang, L. Deng et al., Biologically modified nanoparticles as theranostic bionanomaterials. Prog. Mater. Sci. **118**, 100768 (2021). 10.1016/j.pmatsci.2020.100768

[80] Y. Zhu, P. Xu, X. Zhang, D. Wu, Emerging porous organic polymers for biomedical applications. Chem. Soc. Rev. **51**, 1377–1414 (2022). 10.1039/d1cs00871d 35043817 10.1039/d1cs00871d

[81] X. Zhou, S. Zhang, Y. Liu, J. Meng, M. Wang et al., Antibacterial cascade catalytic glutathione-depleting MOF nanoreactors. ACS Appl. Mater. Interfaces **14**, 11104–11115 (2022). 10.1021/acsami.1c24231 35199514 10.1021/acsami.1c24231

[82] Z. Chen, Y. Sun, J. Wang, X. Zhou, X. Kong, J. Meng, X. Zhang. Dual-responsive triple-synergistic Fe-MOF for tumor theranostics. ACS Nano **17**, 9003–9013 (2023). 10.1021/acsnano.2c10310 37116070 10.1021/acsnano.2c10310

[83] C.E. Wilmer, M. Leaf, C.Y. Lee, O.K. Farha, B.G. Hauser et al., Large-scale screening of hypothetical metal–organic frameworks. Nat. Chem. **4**, 83–89 (2012). 10.1038/nchem.1192 10.1038/nchem.1192 22270624

[84] R.L. Martin, C.M. Simon, B. Smit, M. Haranczyk, In silico design of porous polymer networks: high-throughput screening for methane storage materials. J. Am. Chem. Soc. **136**, 5006–5022 (2014). 10.1021/ja4123939 24611543 10.1021/ja4123939

[85] L.-C. Lin, A.H. Berger, R.L. Martin, J. Kim, J.A. Swisher et al., In silico screening of carbon-capture materials. Nat. Mater. **11**, 633–641 (2012). 10.1038/nmat3336 22635045 10.1038/nmat3336

[86] M. Fernandez, N.R. Trefiak, T.K. Woo, Atomic property weighted radial distribution functions descriptors of metal–organic frameworks for the prediction of gas uptake capacity. J. Phys. Chem. C **117**, 14095–14105 (2013). 10.1021/jp404287t

[87] M. Fernandez, T.K. Woo, C.E. Wilmer, R.Q. Snurr, Large-scale quantitative structure–property relationship (QSPR) analysis of methane storage in metal–organic frameworks. J. Phys. Chem. C **117**, 7681–7689 (2013). 10.1021/jp4006422

[88] P. Raccuglia, K.C. Elbert, P.D.F. Adler, C. Falk, M.B. Wenny et al., Machine-learning-assisted materials discovery using failed experiments. Nature **533**, 73–76 (2016). 10.1038/nature17439 27147027 10.1038/nature17439

[89] D.O. Lopez-Cantu, X. Wang, H. Carrasco-Magallanes, S. Afewerki, X. Zhang et al., From bench to the clinic: the path to translation of nanotechnology-enabled mRNA SARS-CoV-2 vaccines. Nano-Micro Lett. **14**, 41 (2022). 10.1007/s40820-021-00771-8 10.1007/s40820-021-00771-8 PMC8722410 34981278

[90] H. Liu, H. Li, Y. Wang, et al. Machine-Learning Mental-Fatigue-Measuring μm-Thick Elastic Epidermal Electronics (MMMEEE). Nano Lett. **24**, 16221–16230 (2024). 10.1021/acs.nanolett.4c02474 39604089 10.1021/acs.nanolett.4c02474

[91] M.S. Chowdhury, X. Zhang, L. Amini, P. Dey, A.K. Singh et al., Functional surfactants for molecular fishing, capsule creation, and single-cell gene expression. Nano-Micro Lett. **13**, 147 (2021). 10.1007/s40820-021-00663-x 10.1007/s40820-021-00663-x PMC8214653 34146147

[92] G.U. Ruiz-Esparza, X. Wang, X. Zhang, S. Jimenez-Vazquez, L. Diaz-Gomez et al., Nanoengineered shear-thinning hydrogel barrier for preventing postoperative abdominal adhesions. Nano-Micro Lett. **13**, 212 (2021). 10.1007/s40820-021-00712-5 10.1007/s40820-021-00712-5 PMC8523737 34664123

[93] E.J. Corey, W.T. Wipke, Computer-assisted design of complex organic syntheses. Science **166**, 178–192 (1969). 10.1126/science.166.3902.178 17731475 10.1126/science.166.3902.178

[94] X. Ji, L. Ge, C. Liu, Z. Tang, Y. Xiao et al., Capturing functional two-dimensional nanosheets from sandwich-structure vermiculite for cancer theranostics. Nat. Commun. **12**, 1124 (2021). 10.1038/s41467-021-21436-5 33602928 10.1038/s41467-021-21436-5 PMC7892577

[95] D. Zhang, D. Zhong, J. Ouyang, J. He, Y. Qi et al., Microalgae-based oral microcarriers for gut microbiota homeostasis and intestinal protection in cancer radiotherapy. Nat. Commun. **13**, 1413 (2022). 10.1038/s41467-022-28744-4 35301299 10.1038/s41467-022-28744-4 PMC8931093

[96] Y. Wang, L. Lu, G. Zheng, X. Zhang, Microenvironment-controlled micropatterned microfluidic model (MMMM) for biomimetic *in situ* studies. ACS Nano **14**, 9861–9872 (2020). 10.1021/acsnano.0c02701 32701267 10.1021/acsnano.0c02701

[97] C. Kim, S. Hong, D. Shin, S. An, X. Zhang et al., Sorting gold and sand (silica) using atomic force microscope-based dielectrophoresis. Nano-Micro Lett. **14**, 13 (2021). 10.1007/s40820-021-00760-x 10.1007/s40820-021-00760-x PMC8643387 34862935

[98] L. Liu, N. Xiang, Z. Ni, X. Huang, J. Zheng et al., Step emulsification: high-throughput production of monodisperse droplets. Biotechniques **68**, 114–116 (2020). 10.2144/btn-2019-0134 31973559 10.2144/btn-2019-0134

[99] X. Zhang, Y. Wang. AI-recognized mitochondrial phenotype enables identification of drug targets. Nat. Comput. Sci. **4**, 563–564 (2024). 10.1038/s43588-024-00682-9 39174760 10.1038/s43588-024-00682-9

[100] M. Yu, W. Li, Y. Yu et al. Deep learning large-scale drug discovery and repurposing. Nat. Comput. Sci. **4**, 600–614 (2024).10.1038/s43588-024-00679-4 39169261 10.1038/s43588-024-00679-4

[101] F. Han, S. Lv, Z. Li, L. Jin, B. Fan et al., Triple-synergistic 2D material-based dual-delivery antibiotic platform. npg Asia Mater. **12**, 15 (2020). 10.1038/s41427-020-0195-x

[102] B. Zheng, Q. Li, Y. Liu, et al. Microorganism microneedle micro-engine depth drug delivery. Nat. Commun. **15**, 8947 (2024). 10.1038/s41467-024-53280-8 39414855 10.1038/s41467-024-53280-8 PMC11484856

[103] X. Li, Y. Hu, X. Zhang, et al. Transvascular transport of nanocarriers for tumor delivery. Nat. Commun. 15, 8172 (2024). 10.1038/s41467-024-52416-0 39289401 10.1038/s41467-024-52416-0 PMC11408679

[104] J. Ouyang, X. Ji, X. Zhang, C. Feng, Z. Tang et al., *In situ* sprayed NIR-responsive, analgesic black phosphorus-based gel for diabetic ulcer treatment. Proc. Natl. Acad. Sci. U.S.A. **117**, 28667–28677 (2020). 10.1073/pnas.2016268117 33139557 10.1073/pnas.2016268117 PMC7682336

[105] Z. Yang, D. Gao, J. Zhao, et al. Thermal immuno-nanomedicine in cancer. Nat. Rev. Clin. Oncol. **20**, 116–134 (2023). 10.1038/s41571-022-00717-y 36604531 10.1038/s41571-022-00717-y

[106] Y. Yang, X. Wei, N. Zhang, J. Zheng, X. Chen et al., A non-printed integrated-circuit textile for wireless theranostics. Nat. Commun. **12**, 4876 (2021). 10.1038/s41467-021-25075-8 34385436 10.1038/s41467-021-25075-8 PMC8361012

[107] X. Huang, N. Kong, X. Zhang, Y. Cao, R. Langer et al., The landscape of mRNA nanomedicine. Nat. Med. **28**, 2273–2287 (2022). 10.1038/s41591-022-02061-1 36357682 10.1038/s41591-022-02061-1

[108] B. Wang, Y. Li, M. Zhou, Y. Han, M. Zhang et al., Smartphone-based platforms implementing microfluidic detection with image-based artificial intelligence. Nat. Commun. **14**, 1341 (2023). 10.1038/s41467-023-36017-x 36906581 10.1038/s41467-023-36017-x PMC10007670

[109] T. Li, N., Yang, Y. Xiao, et al. Virus detection light diffraction fingerprints for biological applications. Sci. Adv. **10**, eadl3466 (2024). 10.1126/sciadv.adl346 10.1126/sciadv.adl3466 PMC10936869 38478608

[110] S.R. Dabbagh, F. Rabbi, Z. Doğan, A.K. Yetisen, S. Tasoglu, Machine learning-enabled multiplexed microfluidic sensors. Biomicrofluidics **14**, 061506 (2020). 10.1063/5.0025462 33343782 10.1063/5.0025462 PMC7733540

[111] S. Han, T. Kim, D. Kim, Y.-L. Park, S. Jo, Use of deep learning for characterization of microfluidic soft sensors. IEEE Robot. Autom. Lett. **3**, 873–880 (2018). 10.1109/LRA.2018.2792684

[112] X. Huang, Y. Jiang, X. Liu, H. Xu, Z. Han et al., Machine learning based single-frame super-resolution processing for lensless blood cell counting. Sensors **16**, 1836 (2016). 10.3390/s16111836 27827837 10.3390/s16111836 PMC5134495

[113] N. Yang, Q. Shi, M. Wei, Y. Xiao, M. Xia et al., Deep-learning terahertz single-cell metabolic viability study. ACS Nano **17**, 21383–21393 (2023). 10.1021/acsnano.3c06084 37767788 10.1021/acsnano.3c06084

[114] Y. Jiang, C. Lei, A. Yasumoto, H. Kobayashi, Y. Aisaka et al., Label-free detection of aggregated platelets in blood by machine-learning-aided optofluidic time-stretch microscopy. Lab Chip **17**, 2426–2434 (2017). 10.1039/c7lc00396j 28627575 10.1039/c7lc00396j

[115] M.S. Manak, J.S. Varsanik, B.J. Hogan, M.J. Whitfield, W.R. Su et al., Live-cell phenotypic-biomarker microfluidic assay for the risk stratification of cancer patients *via* machine learning. Nat. Biomed. Eng. **2**, 761–772 (2018). 10.1038/s41551-018-0285-z 30854249 10.1038/s41551-018-0285-z PMC6407716

[116] N. Yang, W. Song, Y. Xiao, M. Xia, L. Xiao et al., Minimum minutes machine-learning microfluidic microbe monitoring method (M7). ACS Nano **18**, 4862–4870 (2024). 10.1021/acsnano.3c09733 38231040 10.1021/acsnano.3c09733

[117] M. Yu, W. Li, Y. Yu, Y. Zhao, L. Xiao et al., Deep learning large-scale drug discovery and repurposing. Nat. Comput. Sci. **4**, 600–614 (2024). 10.1038/s43588-024-00679-4 39169261 10.1038/s43588-024-00679-4

[118] K. Jeong, S. Park, D. Park, M. Ahn, J. Han et al., Evolution of crystal structures in GeTe during phase transition. Sci. Rep. **7**, 955 (2017). 10.1038/s41598-017-01154-z 28424509 10.1038/s41598-017-01154-z PMC5430439

[119] S. Park, Y.J. Kang, S. Majd, A review of patterned organic bioelectronic materials and their biomedical applications. Adv. Mater. **27**, 7583–7619 (2015). 10.1002/adma.201501809 26397962 10.1002/adma.201501809

[120] W. Xiao, L. Xin, R. Cao, X. Wu, R. Tian et al., Sensing morphogenesis of bone cells under microfluidic shear stress by holographic microscopy and automatic aberration compensation with deep learning. Lab Chip **21**, 1385–1394 (2021). 10.1039/d0lc01113d 33585849 10.1039/d0lc01113d

[121] A.Y. Mutlu, V. Kılıç, G.K. Özdemir, A. Bayram, N. Horzum et al., Smartphone-based colorimetric detection *via* machine learning. Analyst **142**, 2434–2441 (2017). 10.1039/c7an00741h 28597010 10.1039/c7an00741h

[122] J. Carrete, W. Li, N. Mingo, S. Wang, S. Curtarolo, Finding unprecedentedly low-thermal-conductivity half-heusler semiconductors *via* high-throughput materials modeling. Phys. Rev. X **4**, 011019 (2014). 10.1103/physrevx.4.011019

[123] L. Ward, A. Agrawal, A. Choudhary, C. Wolverton, A general-purpose machine learning framework for predicting properties of inorganic materials. npj Comput. Mater. **2**, 16028 (2016). 10.1038/npjcompumats.2016.28

[124] J. Schmidt, M.R.G. Marques, S. Botti, M.A.L. Marques, Recent advances and applications of machine learning in solid-state materials science. npj Comput. Mater. **5**, 83 (2019). 10.1038/s41524-019-0221-0

[125] M. Zhang, Y. Bai, C. Sun, L. Xue, H. Wang et al., Computational tools for porous materials. npj Comput. Mater. **65**, 462–485 (2022). 10.1007/s11426-021-1171-4

[126] A. Merchant, S. Batzner, S.S. Schoenholz, M. Aykol, G. Cheon et al., Scaling deep learning for materials discovery. Nature **624**, 80–85 (2023). 10.1038/s41586-023-06735-9 38030720 10.1038/s41586-023-06735-9 PMC10700131

[127] N.J. Szymanski, B. Rendy, Y. Fei, R.E. Kumar, T. He et al., An autonomous laboratory for the accelerated synthesis of novel materials. Nature **624**, 86–91 (2023). 10.1038/s41586-023-06734-w 38030721 10.1038/s41586-023-06734-w PMC10700133

