# Correction: Artificial Intelligence-Powered Materials Science

**DOI:** 10.1007/s40820-025-01731-2

**Published:** 2025-04-10

**Authors:** Xiaopeng Bai, Xingcai Zhang

**Affiliations:** 1 World Tea Organization, Cambridge, MA 02139 USA; 2 Department of Materials Science and Engineering, Stanford University, Stanford, CA 94305 USA; 3 Department of Mechanical Engineering, The University of Hong Kong, Hong Kong, 999077 People’s Republic of China; 4 Department of Physics, The Chinese University of Hong Kong, Shatin, Hong Kong, 999077 People’s Republic of China

**Correction to: Nano-Micro Letters (2025) 17:135** 10.1007/s40820-024-01634-8

Following publication of the original article [1], the authors reported that the corresponding author would like to update the email address from xingcai@stanford.edu to drtea1@wteao.com.

Also, the corresponding author’s affiliation can be expanded.

From:

^1^Department of Materials Science and Engineering, Stanford University, Stanford, CA 94305, USA

To:

^1^World Tea Organization, Cambridge, MA 02139, USA

^2^Department of Materials Science and Engineering, Stanford University, Stanford, CA 94305, USA

The corresponding email address and author affiliations have been provided in this Correction. The original article [1] has been corrected.
